# Typical and atypical properties of peripheral nerve allografts enable novel strategies to repair segmental-loss injuries

**DOI:** 10.1186/s12974-022-02395-0

**Published:** 2022-02-28

**Authors:** George D. Bittner, Jared S. Bushman, Cameron L. Ghergherehchi, Kelly C. S. Roballo, Jaimie T. Shores, Tyler A. Smith

**Affiliations:** 1 Department of Neuroscience, University of Texas at Austin, Austin, TX 78712 USA; 2 School of Pharmacy, University of Wyoming, Laramie, WY 82072 USA; 3 Institute for Cellular and Molecular Biology, University of Texas at Austin, Austin, TX 78712 USA; 4 Department of Plastic and Reconstructive Surgery, Vascularized Composite Allotransplantation Laboratory, Johns Hopkins University School of Medicine, Baltimore, MD 21205 USA

**Keywords:** Peripheral nerve allografts, Immunosuppression, Wallerian degeneration, Polyethylene glycol (PEG), T cells

## Abstract

We review data showing that peripheral nerve injuries (PNIs) that involve the loss of a nerve segment are the most common type of traumatic injury to nervous systems. Segmental-loss PNIs have a poor prognosis compared to other injuries, especially when one or more mixed motor/sensory nerves are involved and are typically *the* major source of disability associated with extremities that have sustained other injuries. Relatively little progress has been made, since the treatment of segmental loss PNIs with cable autografts that are currently the gold standard for repair has slow and incomplete (often non-existent) functional recovery. Viable peripheral nerve allografts (PNAs) to repair segmental-loss PNIs have not been experimentally or clinically useful due to their immunological rejection, Wallerian degeneration (WD) of anucleate donor graft and distal host axons, and slow regeneration of host axons, leading to delayed re-innervation and producing atrophy or degeneration of distal target tissues. However, two significant advances have recently been made using viable PNAs to repair segmental-loss PNIs: (1) hydrogel release of Treg cells that reduce the immunological response and (2) PEG-fusion of donor PNAs that reduce the immune response, reduce and/or suppress much WD, immediately restore axonal conduction across the donor graft and re-innervate many target tissues, and restore much voluntary behavioral functions within weeks, sometimes to levels approaching that of uninjured nerves. We review the rather sparse cellular/biochemical data for rejection of conventional PNAs and their acceptance following Treg hydrogel and PEG-fusion of PNAs, as well as cellular and systemic data for their acceptance and remarkable behavioral recovery in the absence of tissue matching or immune suppression. We also review typical and atypical characteristics of PNAs compared with other types of tissue or organ allografts, problems and potential solutions for PNA use and storage, clinical implications and commercial availability of PNAs, and future possibilities for PNAs to repair segmental-loss PNIs.

## Background

The most common neuronal dysfunction in civilian and military life is a traumatic peripheral segmental nerve gap or ablation defect, i.e., a segmental-loss peripheral nerve injury (PNI), as opposed to a simple cut PNI that can be primarily repaired using microsutures through the epineurium/perineurium to oppose the severed proximal/distal nerve ends (neurorrhaphy). Segmental-loss PNIs in more proximal portions of limbs and/or PNIs that involve ablations of  > 5 mm in experimental animal models and humans often have especially poor, if any, restoration of function or coordinated voluntary behaviors, in part because longer gaps lack mechanical guidance and local trophic stimulation for axonal outgrowths [1–4]. This morbidity is a major public health problem. For example, although the estimated incidence of major PNIs in the US is difficult to define, a survey of the National Inpatient Sample for US hospitals representing approximately 20% of all non-federal hospital inpatient admissions identified 138,572 PNIs over 14 years for an average incidence of 67,800 major (segmental-loss, one or more mixed-nerve, more proximal) PNIs per year [5].

Humans (and experimental laboratory animals) with both simple PNIs and segmental-loss PNIs experience [1–4, 6, 7] (1) immediate loss of sensory and motor functions mediated by the denervated target tissues; (2) rapid (3–7 days) Wallerian degeneration (WD) of severed distal axonal segments; and, (3) slow (1–2 mm/day) regeneration by naturally occurring axonal outgrowths from surviving, proximal, axonal ends. This slow outgrowth produces delayed and poor (if any) functional recovery due in part to the frequently encountered long distances required for the slowly outgrowing axons to reach their targets. This slow recovery is also poor due to incomplete and non-specific reinnervation of target tissues and atrophy and/or deterioration of muscle fibers or sensory structures before re-innervation can occur. Severe atrophy and fibrosis from chronic denervation eventually becomes irreversible even if regenerating axons can reach such tissues [8–11].

Immediately restoring and then maintaining normal function is the classical measure of success for transplanted organs [6, 12]. The typical surgical protocol for transplanting a donor *organ* allograft *of non-neuronal origin* (e.g., heart, liver, kidney, lung) is to immediately reconstruct arterial and venous vascular connections to provide oxygenated blood-flow. While there is typically a degree of immunological compatibility matching for non-neuronal organ allografts, the transplants will still evoke a strong immune response that, if not immunosuppressed, will lead to rapid rejection of the donor organ within days [12–15].

The typical protocol for transplanting a non-vascularized donor *tissue* allograft *of non-neuronal origin* (e.g., fascia, cartilage, bone, tendon, skin) is to implant the allograft into the desired recipient tissue bed and expect progressive revascularization by the recipient tissue bed via capillary ingrowth over the course of days to weeks [16–18]. Tissue allografts are almost always performed in a non-immuno-privileged environment, are tissue matched and/or immunosuppressed, typically cannot have a high metabolic rate or burden, and must be sufficiently thin so that they can be revascularized via the wound bed [12]. Restoring and then maintaining normal function over months to years is the typical measure of success for these donor/host allograft tissues of non-neuronal origin [6, 12]. These cell/antigen laden tissue allografts usually evoke a very strong immune response that, if not suppressed, leads to rapid rejection of the donor tissue within days [12, 19, 20]. Non-neuronal tissue allografts that are devoid of cells or have low cellularity typically provide long-term structure and function in place of lost host tissue as a regenerative template (e.g., bone, cartilage, tendon) [21–24].

Peripheral nerve allografts (PNAs) are tissue-type allografts that are transplanted as either vascularized or non-vascularized tissue grafts performed typically without histocompatibility matching between donor and host. Vascularized PNAs have been used for larger diameter and/or longer nerve segments (such as in hand transplantation), while non-vascularized PNAs have been used for smaller diameter and/or shorter segments and may not be immediately revascularized [25–28]. As briefly described below (and in greater detail in later sections of this review), PNAs have some characteristics typical of non-neuronal allograft tissues *and some very atypical* characteristics, including immunological.

Typical to many other types of non-neuronal tissue allografts, vascularized or non-vascularized PNAs are in a non-immuno-privileged environment and evoke a strong immune response. However, PNAs have *atypical* functional and morphological characteristics. That is, the morphologies (e.g., axons, myelin sheaths) and functions (e.g., conduction of action potentials) of PNAs are currently not intended to be maintained immediately upon transplantation, in contrast to non-neuronal tissue (or organ) allografts. Instead, most of functional characteristics and morphological structures of the donor PNA tissues are *atypically* expected to disintegrate within 1–7 days (Wallerian degeneration (WD) and myelin degradation). Immediate restorations of functions/voluntary behaviors are not expected and depend on host axons growing across the PNA to eventually re-innervate denervated host muscle, sensory or organ structures [1, 6, 10, 29–31]. That is, PNAs in current experimental or clinical use are temporary scaffolds or bridges for host axons rather than permanent replacements of host axons. One possible exception is limb transplantation, where nerves transplanted within a limb may maintain donor Schwann cells (SCs) due to their vascularization and ongoing systemic immunosuppression or immunomodulation.

Any re-innervation to restore some original functions in PNAs typically currently occurs from surviving proximal cut ends of *host/recipient* proximal axons via outgrowths at 1–2 mm/day that are gradually remyelinated. Remyelination is accomplished via host SCs and donor SCs that persist if immunosuppression is provided. Furthermore, unlike non-neuronal allografts, restoration of function for PNAs is *atypically* slow (months to years) and often very inadequate. For atypical to non-neuronal allografts, a very inadequate restoration of function after months to years is currently often considered a successful outcome for PNAs [1, 2, 6, 29–33].

This review presents typical and atypical properties of PNAs that enable recently developed novel strategies to repair segmental-loss PNIs, summarizing morphological, biochemical, and immunological data with emphasis on functional/behavioral recovery in experimental animals and humans. We also describe properties of PNAs with other types of allografts, a comparison made by few, if any, other reviews of PNAs in experimental animals and humans. For PNAs in this review, *reinnervation* is the restoration of a nerve supply to a denervated structure by any means at any time after denervation. *Regeneration* is the slow (1–2 mm/day) axonal outgrowth from severed proximal nerve segments that may, or may not, ever successfully innervate a denervated structure. *Nerve axon* refers to the axoplasm and axolemma but does not include the glial sheath; *nerve fiber* refers to the axon and its glial sheath.

## Main text

### Immunosuppression and/or tissue-matching typically required for non-neuronal allografts and PNAs in non-immuno-privileged environments

The non-neuronal and PNA allogenic transplants described above are typically from genetically non-identical donors, are in a non-immuno-privileged environment and are immunologically rejected. These immune responses are almost-always initiated by a local innate inflammatory response that potentiates a subsequent adaptive response [12, 14, 15, 34, 35]. In allograft rejection, including PNAs, macrophages innately promote inflammation and recruit adaptive immune cells, such as T cells, to release inflammatory cytokines, chemokines and reactive oxidative species that exacerbate tissue damage [12, 36, 37]. Most peripheral (non-CNS) tissues contain resident macrophages and many resident dendritic cells (DCs) that are antigen presenting cells (APCs) that enhance T cell activation to reject allografts by direct or indirect allorecognition [38]. That is, major histocompatibility complexes (MHCs) on donor APCs are directly recognized as foreign by host T cells or indirectly allorecognized by host T cells as foreign by minor histocompatibility (mHC) differences. There can be significant MHC and mHC differences in the degree to which different populations of APCs promote allorecognition [39].

The immune response to a skin allograft is coordinated primarily by Langerhans cells (LCs), a DC subtype, followed by other types of DCs, mast cells, and B and T lymphocytes [40–42]. In a skin graft, the graft antigen is presented by resident epidermal LCs, and host and donor LCs and DCs start an adaptative alloimmunity that generates an innate and adaptative immune response. LCs are highly immunogenic as passenger APCs and are strong initiators as host APCs via indirect allorecognition, which is a common feature of DCs resident to boundary tissues, such as skin. These innate and adaptive immune responses are typically greatly reduced or prevented by host/donor tissue-matching and immunosuppression, although LCs may be beneficial in down regulating the alloimmune response [40].

Finally, a few exceptions to rejection of non-neuronal allografts are known and include (1) genetically identical donor/recipient pairs or “isografts” [12], such as the first successful human kidney transplant which was performed between identical twin brothers [43] and (2) allografts in partial or completely immuno-privileged environments such as corneas transplanted to the anterior chamber of the eye, testis, or a mammalian fetus growing in the uterus [12]. These immuno-privileged sites in the body are protected by (a) a physical barrier such as blood–brain barrier in the CNS, peripheral blood barrier in the PNS, efficient blood–retina barrier in the eyes, blood–testis barrier in the testis, and placenta in the mammalian fetus; and/or (b) an inhibitory microenvironment, that inhibits the activity of immunocompetent cells [44]. However, PNAs are not in an immuno-privileged environment and are typically rejected in the absence of tissue-matching and/or immunosuppression [4, 6, 14, 15, 30, 34, 45, 46], although PNAs may have some inherent immunosuppressant properties as described in subsequent sections.

### Segmental-loss PNIs are typically repaired by PN autografts, synthetic conduits or decellularized allografts

If a PNI involves a segmental defect whose proximal and distal cut ends cannot be directly sutured together, regeneration of axons across the defect can be very limited. If there is a more than 0.5 cm long defect in humans (perhaps shorter in smaller animal models), the PN will not regenerate and mechanical assistance is needed to direct regenerating host axons to bridge the gap between the proximal and distal stumps of the host PN [1, 2, 10]. The current “gold standard” for repair/reconstruction of ablation-type PNIs [1, 2, 6] is neurorrhaphy of an ablated segment of several smaller diameter *sensory* nerves taken from less critical locations of the host. Such autografts are cabled in parallel when the diameter of the injured nerve is greater than that of the autografts.

However, a more ideal strategy to repair segmental-loss PNIs would be to use autografts of similar sensorimotor composition, diameter, and axonal number as the injured nerves [6, 46]. The reasons why such matched nerves would promote better repair by regenerating host axons than purely sensory or motor nerves are not completely understood. One possibility is that motor-associated SCs may intrinsically differ from sensory-associated SCs and promote superior regeneration and pathfinding of motor axons [1, 6, 47–49]. Such sensorimotor nerves are not typically feasible for autografting. Hence, autografts of primarily sensory nerves are the experimental and clinical option with the least donor morbidity.

As an alternative to cabled sensory autografts, biodegradable conduits and decellularized PNAs have had some success in repairing smaller length segmental-loss PNIs [50, 51], but lack the complex biological milieu of a live nerve graft. However, conduits and decellularized PNAs have been less effective at promoting peripheral nerve regeneration than sensory autografts for short segmental defects, and cannot be used for long segmental defects, which is a critical unmet need [52]. Much of the field is focused on bioactivating decellularized PNAs and conduits (e.g., cells, growth factors, electrical stimulation) to better simulate the stimulus provided by live nerve grafts [53]. Although these strategies do improve regeneration, they do not yet approach the regenerative capacity of the gold standard autograft, especially for longer segmental PN defects [1, 6, 52–56].

Finally, decellularized PNAs, synthetic conduits, and “gold standard” PN autografts *all* rely on slow (1 mm/day) outgrowth of axons from the severed host proximal nerve stump. Such outgrowths usually produce poor (if any) functional recovery to repair many PNI ablation lesions, especially if the segmental defect is long and if the defect is a great distance away from the innervation targets of the injured axons. Hence, it is problematic that the success of PN repair is often measured not by restoration of lost behaviors (as is the case for other transplanted tissues), but rather by the number of axonal profiles regenerating by outgrowth in the PN segmental gap [1, 7, 32, 33, 57]. Such quantitative counts of axonal profiles often include collaterals from many axons of which few (or none) may actually reinnervate their original targets. Such axonal counts often do not correlate with quantitative measures of functional/behavioral recovery, e.g., SFI, catwalk, toe spread or other assays [1, 4, 7].

### PNAs are potentially better than autografts to repair segmental PNIs

To repair segmental-loss PNIs, PNAs with viable SCs, axons, and other cell types could be better than the current “gold standard”, i.e., cable autograft sensory nerves [1, 2, 6]. *That is, if donor PNAs were ****not**** highly immunogenic, they would have at least 5 advantages over autografts:*

1. PNAs (such as PN autografts) are tissues with viable donor cells that duplicate many complex biological features of an intact PN [46].
2. PNAs can be selected to be predominately motor, sensory, or mixed sensory/motor, whereas autografts are almost-always harvested from exclusively sensory PNs. Regeneration by axonal outgrowth of motor or mixed nerves is superior if mixed grafts are used rather than sensory-only grafts [50, 58–60].
3. PNAs can be more exactly matched to the defect by harvesting the same nerve segment from the donor that was lost in the host. PN anatomical features such as diameter, length, fascicular organization and branching patterns are often important factors in successful reinnervation for regeneration by axonal outgrowth following segmental PN loss [46]. In contrast, the sural, medial antebrachial cutaneous (MABC) or lateral antebrachial cutaneous (LABC) nerves are the most commonly used autografts and often do not match the implant sites in terms of diameter, length, and fascicular organizations. Improper size matching leads to inferior regeneration, fibrosis, and neuromas [61, 62] and fascicular mismatching increases the chances of random target reinnervation and poorer functional outcomes [63, 64]. There is, however, a potential limitation for size matching, because oxygenation and angiogenesis of larger grafts can be limiting factors [65].
4. PNAs can be harvested to match complex nerve structures, such as branch points [11]. No other strategy to repair PN defects includes branch points.
5. PNAs do not require that additional nerves be harvested from the host and thereby do not produce additional host morbidity [1, 6, 7, 30, 32, 33, 46, 66].

### Typical and atypical morphological, functional, and immunological properties of PNAs

#### Typical and atypical morphological and functional properties of PNAs

PNAs are in a non-immuno-privileged environment typical to most non-neuronal allograft tissues, and are not immediately re-vascularized upon transplantation. In the latter peculiarity, they resemble many skin grafts. However, as previously mentioned, PNAs have *atypical* morphological and functional properties compared to other allograft tissues. For example, at least some nerve cells (e.g., dorsal root ganglion (DRG) cells) may have reduced MHCI expression (see following section). Furthermore, PNAs morphologically consist largely of donor SCs and their myelin sheaths and axons no longer connected to their cell bodies, i.e., anucleate segments of membrane-bound cytoplasm. Most atypically, the morphology and function (conduction of action potentials) of nerve fibers are not intended to be maintained immediately upon transplantation as is the case for other allograft tissues, but rather via WD are expected to disintegrate within 3–7 days [1, 6, 14, 15, 46].

The SC connective tissue sheaths of PNs can act as morphological and chemoattractant guides for somatic or autonomic host axons to possibly re-innervate denervated target tissues, such as muscle fibers and sensory end organs. This possible (but often infrequent) re-innervation occurs from surviving proximal cut ends of host proximal axons via outgrowths at 1–2 mm/day and does not require long-term survival and function of donor allogenic cells [1, 6]. In contrast, the functions of most non-neuronal allografts typically depend upon their maintaining viable donor allogenic cells [12, 67].

#### Typical and atypical immunological properties of PNAs

The initial (within hours to days) innate immune response of PNAs to axonal transection and subsequent WD within 3–7 days is initially mediated primarily by (1) donor macrophages, a cell type and response typically seen in non-neuronal allografts [12] and (2) SCs, a cell type atypical to PNAs [15, 68, 69]. SCs are a specialized PN cell type derived from neural crest cells whose embryonic origins are somewhat unique [70].

**Fig. 1:**
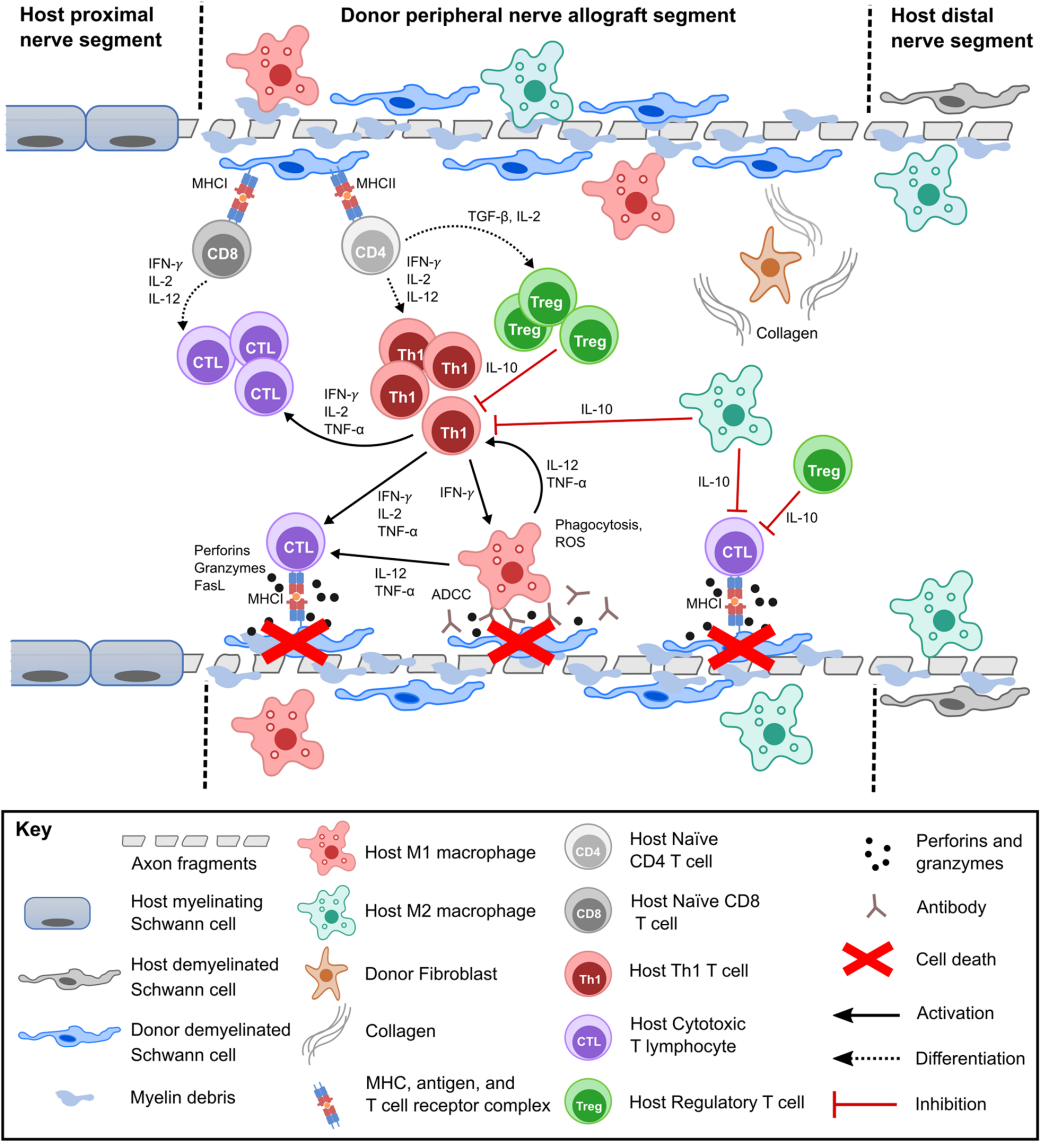
Diagram showing immunological responses to donor PNAs. After about 7 days post-engraftment, donor demyelinated Schwann cells presenting donor antigens via MHCI are recognized by host CD8 T cells within the peripheral nerve allograft segment. Over a period of several weeks, activated CD8 T cells clonally expand and differentiate to produce populations of cytotoxic T lymphocytes (CTLs) when stimulated by IFN-γ, IL-12, and IL-2. CTLs find antigen-bound MHCI molecules on donor cells and then induce donor cell death (shown as red X’s) in donor cells via perforins, granzymes, and FasL. CD4 T cells activated by donor antigens on MHCII on either host or donor antigen-presenting cells differentiate into T helper 1 (Th1) cells and regulatory T cells (Tregs), among other T cell subtypes, depending on whether the CD4 T cells are stimulated by IFN-γ or TGF-β, respectively. Th1 cells secrete cytokines such as IFN-γ, IL-2, and/or TNF-α that stimulate survival and pro-inflammatory activation of CTLs and macrophages. Classically activated M1 macrophages contribute to donor Schwann cell death via release of reactive oxidative species (ROS), phagocytosis, TNF-α, and Antibody-Dependent Cellular Cytotoxicity (ADCC). M1 macrophages augment the activation of Th1 cells and CTLs via IL-12 secretion. Tregs and alternatively activated M2 macrophages secrete the anti-inflammatory cytokine IL-10 which regulates and suppresses pro-inflammatory functions in Th1 cells and CTLs. Fibroblasts produce large amounts of collagen throughout the wound healing process

SCs differentiate into activated phenotypes that degrade and phagocytose myelin [14, 15, 69, 71]. Activated SCs initiate signaling cascades that rapidly recruit other immune cells such as host macrophages that help remove degraded axonal and myelin debris, a morphological and functional characteristic peculiar to PNAs (Fig. 1). Donor SCs and endothelial cells are the primary rejection targets in PNAs and present allogenic major histocompatibility complex I and II (MHCI and MHCII) cell surface proteins which themselves serve as alloantigens [14, 15, 34, 72] (Fig. 1). Studies investigating the macrophage response to nerve crush and single transection injuries in mouse models demonstrated that “alternatively-activated” M2 macrophage subsets reduce the inflammatory response via Interleukin 10 (IL-10) secretion, stimulate angiogenesis via Vascular Endothelial Growth Factor A (VEGF-A) secretion, and phagocytose myelin debris in concert with SCs [59, 73]. Fibroblasts secrete growth factors and form much of the extracellular matrix that mechanically and chemically guides axons and SCs [62, 63, 74] (Fig. 1).

Days to weeks after a segmental-loss PNI, macrophages stimulate SCs and other immune cells to complete myelin breakdown and begin to direct axonal regeneration [69]. Many of these typical adaptive immune responses of PNAs at 5–20-days post-injury are also typically reported for non-neuronal allografts [12]. For example, rejections of PNAs and other non-neuronal allografts are mediated by donor antigen-presenting cells (APCs), mostly macrophages, that remain within the graft and/or migrate to secondary host lymphoid tissues to activate host T cells [12]. Intact PNs, and PNAs *atypically* have very few resident DCs relative to other allografts [46].

Migrations of alloreactive effector T cells typically induce rejection of PNAs and other types of allografts by direct allorecognition, typically within 2 weeks. Acute PNA rejection is mediated, in part, by cytotoxic CD8^+^ and/or helper CD4^+^ T cells that react to foreign MHCs and foreign antigens presented by MHCI and MHCII moieties [75] (Fig. 1). Direct allorecognition stimulates host responses via recognition of polymorphic non-self-MHC proteins on donor cells by T cell receptors (TCRs) [76, 77]. Rejection also typically depends on indirect allorecognition of minor histocompatibility antigen (mHA) differences. These alloantigens are incorporated and presented by host APCs to activate host T cells [78, 79]. Hence mHA differences can evoke an alloresponse even when MHCs are matched [80]. Activation of T cells by either MHC or mHA differences can occur locally in an allogenic graft or remotely within a lymph node outside of the graft, where the T cells from lymphatic nodes chemotax to the graft and induce rejection [81, 82].

However, the immune response of PNAs is *atypical* in several aspects compared to non-neuronal allografts. For example, without any immunosuppression, donor allogenic SCs are viable in PNAs at 14 days after transplantation and form most of the glial connective tissue bridges between donor and host nerve segments that can guide regenerating axons [46]. These donor SCs also migrate extensively into host PNA tissue. At 14–28-days post-transplantation, the number of donor SCs sharply declines as they are presumably eliminated by host T cells. However, a small number of donor SCs persist, suggesting that these donor SCs evade much of the host immune response and adapt to their environment largely composed of host-derived cells [46]. Furthermore, the infiltration of immune cells into PNAs and PN autografts are similar, having (**1**) approximately equal quantities of CD4 T cells at all-timepoints, and (**2**) CD8 T cells equal in PN autografts and PNAs at 7 and 14 days post-implantation, albeit higher in PN autografts at 3 days. In contrast to T cells, macrophages are 46%, 47% and 148% higher, respectively, in PNAs at 3, 7 and 14 days compared to PN autografts, but equivalent to PN autografts at 28-days post-transplantation [46].

### Possible endogenous reasons and mechanisms for reduced PNA immunological responses

Since PNAs are not in an immunoprotected environment, a host would typically be expected to acutely reject a donor PNA via an immunological cascade similar to other allogeneic tissues or organs. Direct allorecognition would be evoked by MHC incompatibility between passenger APCs interacting with TCRs of infiltrating host lymphocytes. Indirect allorecognition would occur as host APCs take up and present donor mHA antigens. Both direct and indirect pathways lead to activation and proliferation of graft-specific T cells that would progressively attack and eliminate donor cells with incompatible MHCs or displaying immunoreactive mHAs.

However, the immune response of PNAs may be somewhat immuno-privileged, because:

1. PNAs are surrounded by an epineurium that may slow immune cell infiltration. The epineurium consists primarily of type I collagen deposited by fibroblasts, forming a basement membrane that contains the vascular supply of intact PNs whose vascular endothelial cells have many tight junctions [83]. The connective tissue and vasculature form the peripheral blood nerve barrier (BNB) that controls the transport of chemical substances and cells from the vascular system into PNAs [83] to regulate the immune response [84]. The infiltration of immune cells and macromolecules is less permissive under basal conditions and more selective during the immune response to a PNA [85]. Furthermore, immune cells infiltrate the PNA and host PN tissue at the donor–host boundaries and not along the entire PNA [46]. This infiltration may contribute to the more gradual immune response to a PNA. However, host T cells are present in PNAs, as typically found in non-neuronal allografts.
2. PNAs have baseline expressions of MHC and mHA that are lower than other tissues and do not increase as much after transplantation compared to other tissues [86, 87]. PNA MHCs also display fewer mHAs. For example, donor skin harvested from allogenic mice that lacked either class I or class II MHC glycoproteins had a slower rejection rate compared to donor skin expressing both MHC classes. However, complete rejection eventually occurred in skin transplants that lacked either MHCI or MHCII antigens. In contrast, when these experiments were replicated with allogenic PNAs, rejection did not occur for PNAs that lacked either MHCI or MHCII glycoproteins. Part of this reduced expression in PNAs might be because intact PN neurons expressed little to no MHC I and, therefore, may be a relatively immuno-privileged cell type [88].
3. PNAs have differences in APC location, abundance and activity compared to other allograft tissues. APCs such as macrophages and DCs are essential for both direct and indirect allorecognition, where direct allorecognition is the more rapid process and is primarily mediated by passenger APCs interacting with host T cells [82]. PNAs lack lymphoid follicles and have fewer DCs as do intact PNs when compared to many other transplanted tissues [6]. PNs have many macrophages, but these appear to skew toward M2 polarization in PNIs that display fewer antigens and are less likely to initiate an acquired immune response. The relative scarcity of many passenger immune cells within PNAs might explain why donor SCs initially persist after transplantation, but are eventually eliminated via mHA differences/indirect allorecognition [46].

### Typical and atypical bioengineered mitigations of the host immune responses to donor PNAs

Similar to other allograft tissues described in previous sections, innate and adaptive rejections of PNAs are typically greatly reduced or prevented by chronic treatment with systemic immunosuppressants. These drugs are often toxic and have side-effects, such as opportunistic infections, increased risk of diabetes, malignancy and renal failure [89, 90]. For example, cyclosporine (CsA) and tacrolimus (FK506) have become widely used for immunosuppression of transplanted organs and tissues. Both drugs inhibit serine/threonine phosphatase calcineurin, thereby preventing calcineurin from dephosphorylating the nuclear transcription factor of activated T cells (NFAT) [91]. Dephosphorylation of NFAT activates T cells to proliferate and produce cytokines that enhance the acquired immune response [91].

In contrast to results for most other allogenic organ or tissue allografts that are completely rejected and fail without immunosuppression, host axonal regeneration through donor PNAs without any immunosuppression can be quite robust and permanent. This result has been noted in species ranging from rodents to primates, with regeneration measured by a variety of morphological, histological, electrophysiological and functional outcome measures [92]. Furthermore, PNAs only require temporary systemic immunosuppression. Once axons either regenerate through the PNA or reinnervate target tissues, immunosuppression can be discontinued without a loss of function [92].

### Two novel strategies to repair segmental-loss PNIs by PNAs

Most recently, two innovative conceptual approaches and detailed protocols have been independently developed that mitigate the immune response to PNAs and significantly enhance functional/behavioral recovery following segmental-loss PNIs.

**Fig. 2:**
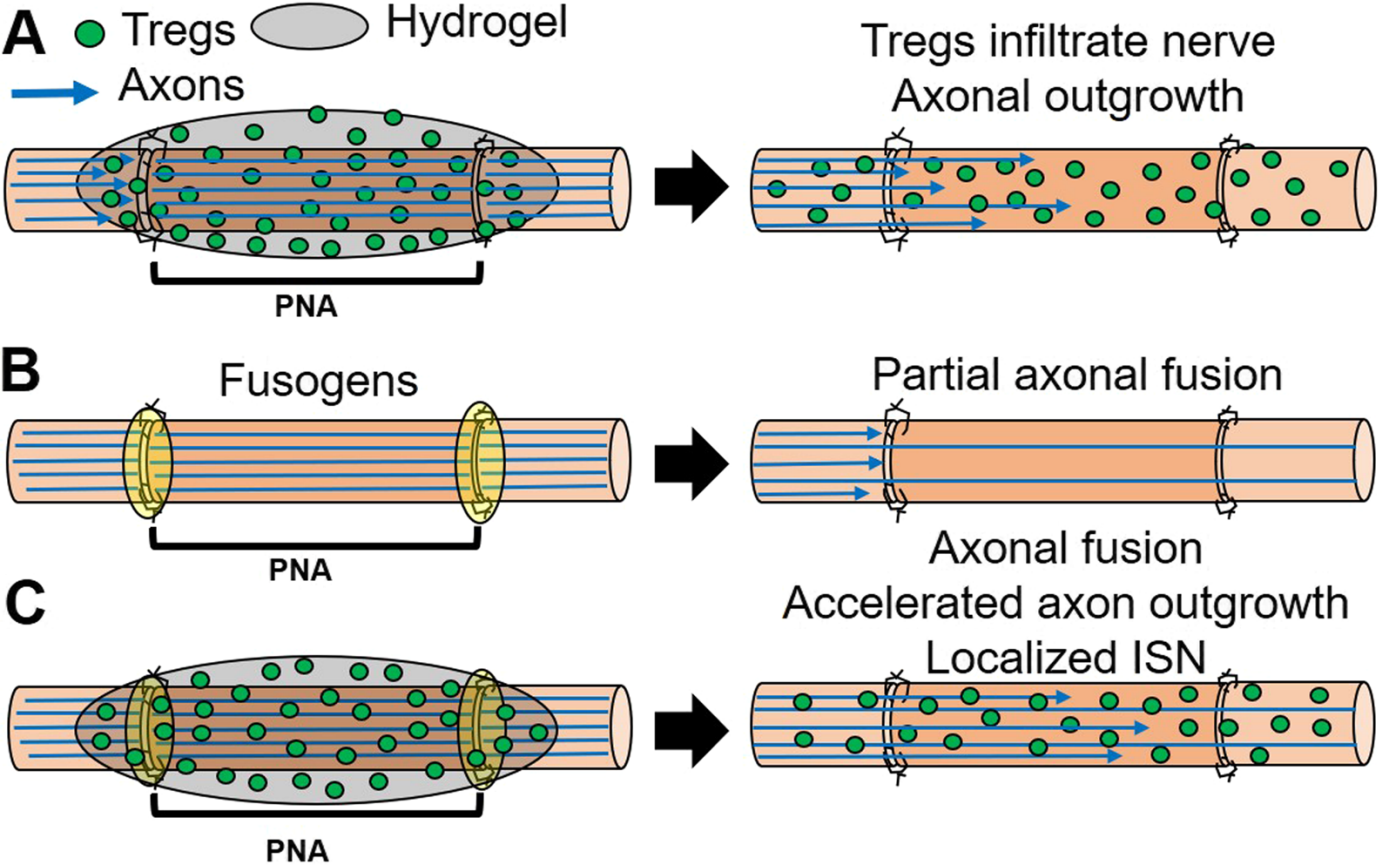
Strategies to use PNAs for regeneration of segmental PN defects. **A** Concept of localized immunosuppression, delivering Tregs that infiltrate graft, suppress the host immune response and support axonal extension. **B** Concept of axonal fusion across a PNA, restoring immediate electrical conductance. **C** Combination of axon fusion and localized immunosuppression that restores immediate conductance and protects the graft from the host immune response during critical periods and re-innervation based on axonal regeneration by outgrowth

The first novel strategy is to deliver immunosuppressive Tregs only to the PNA at the time of its implantation using a poly(ethylene glycol) (PEG) norbornene (PEGNB) hydrogel as the Treg delivery vehicle. As the hydrogel degraded (Fig. 2A), the Tregs chemotaxed and infiltrated the PNA, locally suppressed the host immune response to the allograft, and enabled full regeneration equal to the sensorimotor matched autograft in a rat 2 cm defect without any additional immunosuppression [93]. This technology localizes immunosuppression only to the PNA, circumventing the health risks of systemic immunosuppression, and induces robust axonal outgrowth.

The second novel strategy uses a plasmalemmal fusogen (polyethylene glycol, aka PEG) to morphologically connect (join) the axolemma and axoplasm of some donor/host (recipient) axons in a PNA, thereby immediately restoring conductance of action potentials across the PNA (Fig. 2B). The PEG-fusion protocol for PNAs consists of neurorrhaphy combined with localized application of a well-defined sequence of four pharmaceutical agents in solution—one of which has a high concentration of the membrane fusogen PEG [7, 32, 33, 57]. This PEG solution is directly applied to, and immediately and non-selectively fuses/joins, opened ends of severed host and donor *viable* axons closely opposed by neurorrhaphy at both ends of the PNA. This strategy to immediately (somewhat-to-very-randomly) re-innervate denervated distal sensory and motor targets is qualitatively different than any other experimental or clinical strategy currently used to repair singly cut or segmental-loss PNIs.

These technologies and their clinical implications are discussed in more detail in following sections. A combination of these two technologies *might* be particularly efficacious (Fig. 2C).

### Typical and atypical properties of PNAs enable a novel strategy of localized immunosuppression

Use of PNAs with viable SCs and other donor cells to repair segmental PNIs in host organisms necessitates immunosuppression of the host. For example, a clinical case treated severe right intercostal neuralgia with a PNA and immunosuppression consisting of FK506, azathioprine, and prednisone administered systemically from 3 days prior to the surgery until 14 months after surgery [176]. This regimen produced significant nerve regeneration, but a delay in wound healing due to the systemic immunosuppression. In one such study in sheep with PNAs, all animals that received systemic immunosuppression experienced severe opportunistic infections that caused mortality and premature conclusion of the study [94]. Opportunistic infections are a serious concern for those with traumatic injuries, the most common cause of segmental PNIs, and can rapidly propagate in immunosuppressed patients. If the pathogen cannot be effectively treated, diminished graft and even patient survival are consequences [95]. Cancer is another risk of chronic immunosuppressive therapy, where rates of cancers in the immunosuppressed patients are significantly greater for more at least 32 different malignancies than the general population [96–98]. Immunosuppressive drugs are also toxic over time to normal tissues, particularly renal tissue [97, 98]. Finally, administration and clinical monitoring of immunosuppressive therapy is very expensive [99]. All of these issues create a balance between therapeutic benefit and risks/cost, where the balance is acceptable for organs that preserve life but less so for quality-of-life improvements, such as PNAs.

The transplantation community has long sought better ways to mitigate allograft rejection via tolerance or immunosuppression that could be localized to the graft. Immunosuppression localized to the allograft might prevent its rejection, but allow the immune system to be fully functional elsewhere. Drug-based localized immunosuppression is complicated by the systemic interaction of immune cells. Direct and indirect allorecognition can occur to activate the host immune cells both local to the graft and at distant lymphatic centers [100]. Calcineurin inhibitors primarily prevent activation of host immune cells rather than inhibiting their activity once already activated. Localized release of these drugs in the vicinity of the PNA would have little effect on host immune cells that were activated outside the localized zone of drug-based immunosuppression. Such cells could likely still infiltrate the grafts and mediate rejection even in the presence of calcineurin inhibitors. An additional complicating factor for PNAs is graft length and location of the PNI. Localized drug release technologies would need to provide immunosuppression for different periods of time, depending on how long it takes for axons to regenerate across the graft and/or reinnervate target tissues [6, 46].

Some atypical properties of PNAs may enable or enhance localized immunosuppression. As discussed previously, PNAs without any immunosuppression still exhibit substantial axonal regeneration and PNAs that undergo temporary systemic immunosuppression achieve functional outcomes equal to continuous immunosuppression [94, 101]. Therefore, complete PNA immune tolerance probably isn’t necessary, and the eventual rejection of a donor PNA does not appear to harm regenerated host axons [6, 46].

A localized cell-based approach to PNA immunosuppression might be a viable alternative to immunosuppressive drug release localized to the PNA. As one possibility, Tregs are an immunosuppressive sub-population of CD4^+^ lymphocytes that (1) modulate acquired immune responses and maintain tolerance to self-antigens, (2) have angiogenic properties, (3) can be isolated from peripheral blood, (4) are equally effective when allogenic to the host, and (5) have been safely and successfully used in several clinical trials [102–106]. Tregs chemotax to activated APCs and effector T cells inhibit these cells through cell–cell contact and paracrine signaling [102, 107]. Importantly, immunosuppression of Tregs prevents activation of immune cells and inhibits immune cells that are already activated. In addition, the mitogens and cytokines that promote Treg proliferation, chemotaxis and functional immunosuppression are secreted by activated Tregs that could be localized to PNAs. Consequently, Treg abundance and activity is regulated by the presence of the effector cells the Tregs suppress. This may be an advantage of cell-based systems, where the longevity of the localized immunosuppression via cells is autoregulated.

Animal studies and clinical trials with Tregs for other indications such as grafts vs host disease or liver transplantation rely on systemic delivery of Tregs, requiring billions of Tregs for a single infusion [108]. Localized Treg delivery only to a PNA would ensure that sufficient quantities of Tregs are placed where needed, eliminate or reduce problems of systemic Treg delivery such as off-target engraftment or reduced general immunity, and lower the quantity of Tregs needed from billions to a few million [109]. A method for localized release of Tregs has recently been obtained by a hydrogel delivery vehicle composed of PEGNB [6, 46, 93]. The PEGNB carrier was used to preserve Treg viability and to promote Treg release at the time when the Tregs would chemotax to the host immune cells infiltrating the PNA. PEGNB hydrogels were optimized to maintain Treg viability in the absence of mitogens (IL-2, αCD28), where 70% of Tregs remained viable within PEGNB after 14 days compared to 36% viability for Tregs cultured under standard tissue culture conditions in Treg media, but without mitogens. PEGNB was optimized to degrade by 14 days, so that Tregs would be released from the PEGNB during the time when host immune cells are known to be infiltrating and engrafting within the PNA [93]. In vitro cell release experiments showed that 85% of Tregs initially encapsulated into the PEGNB were released as viable cells over the 14-day period that the PEGNB degraded.

To test the effects of localized release of Tregs to PNAs, allogenic Tregs isolated and expanded from the spleens of from GFP Sprague Dawley (SD) rats were implanted around a branched 2 cm SD GFP donor PNA placed in the segmental-loss sciatic nerve gap of host Lewis rats [93]. Tregs were diluted to 1 × 10^6^ per 100 µl of the PEGNB, with approximately 450 µl of PEGNB encapsulating the PNA within the tissue cavity. GFP-labeled Tregs infiltrated and spread throughout the PNA and adjoining host nerve tissue in a pattern consistent with host immune cell localization to the PNA. At 21 days after implantation, which was the last timepoint Treg localization was assessed, GFP Tregs were abundant and uniformly distributed throughout the PNAs and adjoining host nerve tissue. It is not known how long the Tregs remained in the PNAs past 21 days, but Treg number may gradually decline due to exhaustion and/or a reduction in IL-2 or other Treg survival factors secreted by host immune cells.

Some Tregs were observed in the red pulp of the spleen 3 and 7 days after implantation and were gone by 14 days. This result suggested that some Tregs escaped the PEGNB and entered the systemic circulation, but did not accumulate in the spleen, which is a common off target engraftment site for cells of hematopoietic lineage. Quantification of host CD4^+^ helper T cells in the grafts via densitometry at different timepoints in Treg-treated PNAs showed significant reductions in host CD4 + T cells, indicating that the Tregs functionally suppressed the host immune response [93].

Compound muscle action potential (CMAP) amplitudes were used to quantify muscle electrical response as a standard metric of motor axon reinnervation in the clinic and animal studies [110]. CMAP amplitudes showed that PNAs with localized Tregs stimulated recovery of muscle electrical activity equal to control PN autografts using a sensorimotor-matched nerve. The PN autograft was sutured back into the defect in the exact same configuration (not reversed) within 15 min of its excision to provide the best-known possible environment for axonal regeneration and muscle reinnervation. The CMAP amplitude and latency of both the tibial and peroneal branches of the sciatic nerve were similar for PNAs with Tregs and the “gold standard" PN autografts [93].

While CMAP amplitude of PNAs with Tregs were equivalent to those recorded from PN autografts at similar post-lesion times, toluidine-blue stained cross sections of the nerves showed distinctive morphological differences for PNAs with Tregs compared to PN autografts. Cross sections assessed in the PNA ~ 0.5 cm distal to the proximal–distal suture point showed a higher total axon density for PNAs with Tregs compared to the autografts, but the axons were condensed into a smaller area within the PNA compared to more spread out in the autografts. This curious morphology was noted for every single animal that received the PNA with Tregs [93]. The implications of this distinctive morphology and the reasons for it are an interesting unanswered question.

While these are promising results for a new methodology of localized Treg delivery for localized immunosuppression [6, 93], there are many questions that remain to be addressed. For example (1) Do PNAs with Tregs promote recovery similar to autografts with respect to behavioral metrics of functional regeneration? (2) Is immunosuppression localized to PNAs or is there off-target Treg engraftment in other tissues (e.g., lung, lymph nodes)? (3) Do animals with Treg suppressed PNAs mount an appropriate immune response to a pathogen challenge elsewhere in the organism? (4) Are sub-populations of Tregs more effective and do other cell types (e.g., myelinating SCs) have immunosuppressive properties?

### Typical and atypical properties of PNAs enable a novel strategy of PEG-fusion to repair segmental-loss PNIs in the absence of tissue-matching or immunosuppression

#### PEG-fusion protocol and rationale

Bittner et al. (1990) [111] originally developed PEG-fusion as an experimental phenomenon to repair invertebrate giant axons. This laboratory subsequently developed protocols that enabled fusion of crushed mammalian axons ex vivo and in vivo following PNIs [112–114]. PEG-fusion was further improved to repair single transections [113, 115, 116]. This technology has more-recently been extended to PEG-fuse PNAs to repair segmental-loss PNIs [4, 15]. As outlined in Table 1, the PEG-fusion protocol to repair PNAs consists of five steps [34]: (1) Application of calcium-free hypo-osmotic saline to freshly trimmed host and donor nerve ends to expel vesicles and increase axoplasmic volume. (2) Direct application of 1–2 drops of the anti-oxidant methylene blue (MB) 0.5–1% in ddH_2_0 to reduce accumulation of intracellular vesicles, preventing partial collapse of axonal ends. (3) Neurorrhaphy to oppose host and donor nerve segments. (4) A high concentration (50% w/w in ddH_2_O) of the membrane fusogen PEG directly applied sequentially to the coaptation site of the proximal end of the PNA and then to its distal end. PEG immediately (seconds to minutes) and non-selectively fuses/joins host/donor axons at the proximal and distal ends of a PNA. (5) A fourth isosmotic solution contains calcium that seals any remaining small axolemmal holes. This PEG-fusion protocol typically restores through-conduction of action potentials across a segmental-loss PNI [33]. Note that none of these solutions are toxic or produce distress in the host animal.

**Table 1:** PEG-fusion protocol and rationale to immediately repair (join) cut axonal ends

Protocol Steps #1–5	Completely sever and trim nerve ends	Prepare nerve ends for neurorrhaphy and PEG repair/fusion
1. Priming Solution #1	Irrigation of surgical field with hypotonic Ca^2+^-free saline for 1–2 min	Increase axoplasmic volume. Open cut axonal ends. Expel intracellular membrane-bound vesicles/organelles
2. Protection Solution #2	Administer 0.5–1% methylene blue (MB; an antioxidant) in distilled water for 1–2 min to opened cut ends	Prevent formation of intracellular vesicles/organelles that interfere with PEG-fusion of cut ends and can seal-off each apposed cut end rather joining/fusing them
3. Co-apt cut nerve ends	Perform neurorrhaphy	Provide mechanical strength to epineurium to prevent PEG-fused axons from pulling apart. Closely appose cut axonal ends
4. PEG-fuse many axons Solution #3	Apply 50% w/w 3.35 kDa PEG in ddH_2_O for 1–2 min to the coaptation site	Remove bound cell water to induce closely apposed, open, axonal membranes to non-specifically fuse
5. Membrane repair Solution #4	Irrigation of coaptation site with isotonic Ca^2+^—containing saline	Induce vesicle formation to plug/seal any axolemmal holes after PEG-induced annealing of open cut axonal ends

#### Overarching compound hypotheses to account for morphological, functional and immunological results of PEG-fused PNAs

**Fig. 3:**
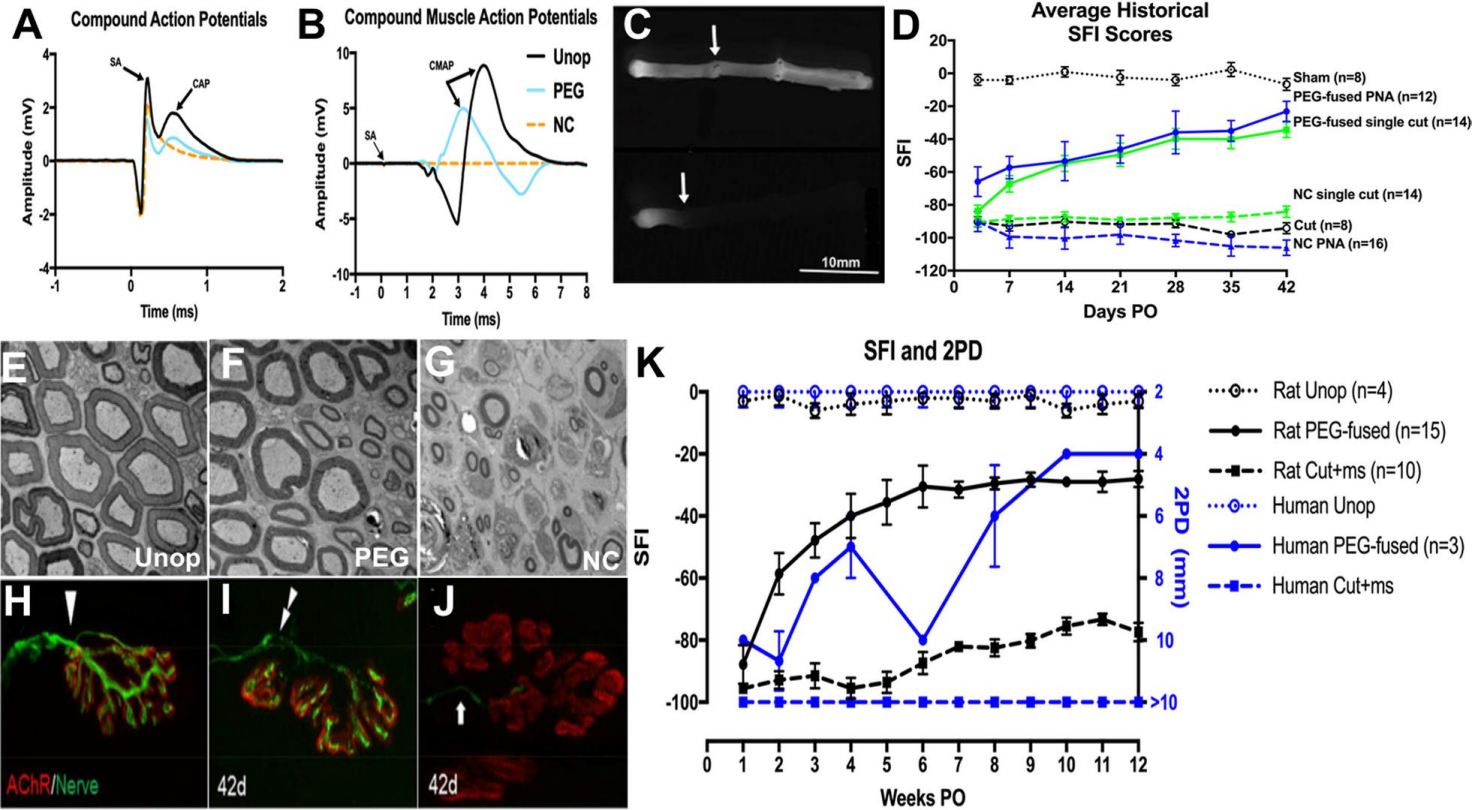
PEG-fusion of PNAs from SD donors to SD (outbred) recipients promote axonal fusion and accelerated recovery after segmental PNI. **A** CAP (mV) recordings of intact sciatic nerve (Unop: black solid line) stimulated near the spinal cord and recorded distal to the PNA after ablating a 1 cm segment, insertion of a slightly longer donor segment without (NC: orange dashed line) or with PEG-fusion (PEG: blue solid line). SA = stimulus artifact. CAP arrow = peak amplitude. **B** CMAP recordings using same stimulating protocols as CAPs, but recorded from the tibialis anterior muscle. **C** Intra-axonal dye diffusion of Texas Red at 1-day PO in a NC (top) or PEG-fused (bottom) sciatic nerve. Arrows point to proximal cut end of host sciatic nerve microsutured to proximal end of donor PN. **D** SFI scores vs post-lesion time from rats that are Sham Controls, NC single cut PNAs, and PEG-fused single cut or PNAs. **E**, **H** Unoperated, **F**, **I** PEG-fused and **G, J** NC TEM, **E–G** and IHC **H–J** images of distal sciatic nerves and NMJs at 42-day post PEG-fusion of PNAs. **K** PEG-fusion nerve repair improved outcomes and speed of nerve recovery in the clinical setting as assessed by average MRCC score. The time course and extent of the clinical recovery of two-point discrimination (2PD) is similar to that reported for SFI behavioral recovery in rats when both are plotted on the same graph

*PEG-fusion of PNAs is a unique technology compared to all other allograft transplant procedures, because PEG-fusion repairs many transected cells (i.e., axons) that would otherwise undergo WD in both graft PNA and host axons distal to the PNA* (Figs. 2, 3). That is, PEG-fusion of PNAs immediately repairs host cells in a donor allograft and does *not* necessarily maintain any donor cells as opposed to restoring vascular supply to other types of donor allografts (e.g., hearts, kidneys) to maintain their donor cells in the host organism. Unique to PEG-fused PNAs, many donor and host axons are almost-certainly maintained by host proteins and, therefore, resemble a chimeric donor–host construct that elicits attenuated pro-inflammatory and enhanced anti-inflammatory responses from host immune cells compared to traditional PNAs without PEG fusion [4, 14, 15, 33]. That is, PEG-fused PNAs are immune-accepted due to reduced innate and adaptive inflammatory immune responses (and/or increased anti-inflammatory responses) that are produced by immediate repair of many host and donor PEG-fused axons that all receive host proteins. PEG by itself may also have immunoprotective effects in the PEG-fusion protocol. Furthermore, the immune-acceptance persists for at least 4 months, perhaps permanently, in rats [4, 14, 15].

### Consequently, in contrast to other techniques that do not repair transected axons

**Fig. 4:**
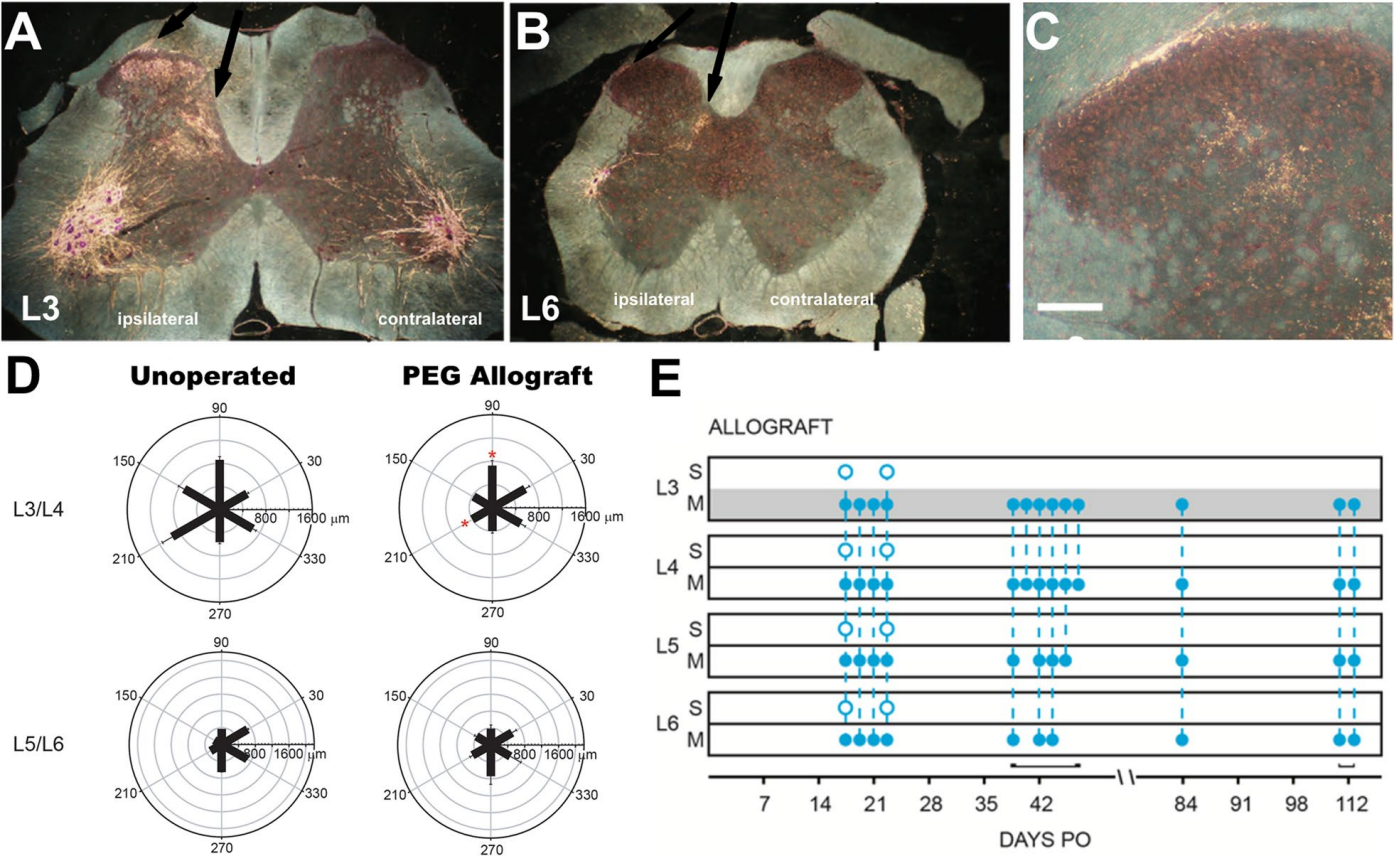
Location of BHRP-labeled motoneurons and primary sensory afferent spinal projections. **A**–**C** Darkfield digital micrographs of transverse hemisections through lumbar spinal cord injection of Botulinum–toxin conjugated HorseRadish Proxidase (BHRP) in the tibialis anterior muscles. **A** BHRP injection into the TA muscle labeled control, undamaged, motoneurons in the L3 spinal segment in the contralateral side (Unoperated). BHRP injection into the ipsilateral TA after PEG-fusion repair of segmental-loss sciatic nerve PNI often labels original appropriate motoneurons in L3 segments **A** as well as atypical, inappropriate motoneurons in other spinal segments **(B**), e.g., L6 and sensory afferents (black arrows)**. C** Anomalous sensory afferent terminal labeling in dorsal horn lamina I–V of L3 through L6 segments of PEG-fused Allograft animal at 17 days PO. Scale bar = 100 μm. **D** Polar plots of total length of dendritic material divided into radial sectors for measure of motoneuron dendritic distribution in 6 bins of 60° each. Bar lengths represent means ± SEM. * indicates significantly different from Unoperated Controls (*p* < *0*.*05*). **E** Patterns of motoneuron and sensory labeling in spinal cord segments L3–L6 after injecting BHRP into the TA in PEG-fused Allograft animals. S: Sensory, M: Motor. Gray L3 M bar indicates normal location of TA motoneurons in Unoperated Controls. Blue open circles indicate location of sensory terminal label; filled circles indicate location of BHRP-labeled motoneurons. Vertical dashed lines represent labeling across multiple lumbar sections within individual animals sampled at a given PO time

1. PEG-fusion of PNAs immediately (seconds to minutes) morphologically (Fig. 3C) and functionally repairs their transected axons so that they conduct axon potentials from spinal cord to muscle contraction at functional NMJs (Fig. 2A, B). PEG-fusion induces *immediate* reinnervation of many otherwise-denervated target tissues [4, 14, 15, 30, 32, 33, 66]. This morphological and functional continuity prevents WD (Fig. 2E–J), the immediate or long-term rejection of PNAs, and most muscle atrophy (Table 2). However, while muscles contract when activated by motoneurons, many (or most to almost all) of these fusions produce randomly connected host and donor axons (Fig. 4) that produce spastic (uncoordinated) activation of distal muscle masses (Fig. 3D) and do not immediately restore their original voluntary, coordinated behavioral, muscle activities or sensory perceptions (Fig. 3D).
2. PEG-fusion of PNAs subsequently typically restores lost voluntary behaviors and sensory perceptions within weeks (Fig. 3D), sometimes approaching measures of voluntary behaviors exhibited by Unoperated Control rats [4, 14, 15, 30, 32, 33, 66].
3. *Axons that are not initially successfully PEG-fused can naturally regenerate by outgrowth from surviving proximal stumps and typically take at least 6*–*8 weeks to re-innervate denervated target tissues following sciatic nerve segmental-loss PNIs* [4, 32, 33; Table 2].

**Table 2:** Summary of allograft axonal morphometric data at 7–42-days PO

Days PO	Animal, group	SFI	Avg axon diameter			Avg g-ratio			Axons/10,000 µm^2^			% Axons > 3µm			Axons >3µm /10,000µm			MFA	MFI (sol)
			Prox	Graft	Dist	Prox	Graft	Dist	Prox	Graft	Dist	Prox	Graft	Dist	Prox	Graft	Dist	(µm^2^)	(%)
Unop Avg	*n* = 3	-4	3.95 ± 1.74	[3.88]	3.82 ± 1.64	0.62 ± 0.06	[0.62]	0.61 ±0.06	210	[201]	191	64%		63%	133		118	2710 ±710	100%
7d PO																			
7d PEG Avg	*n* = 3	-92																	71%
7d NC Avg	*n* = 4	-97		0	0		0	0		0	0		0%	0%		0	0		0%
21d PO																			
21d PEG Avg	*n* = 5	-33	3.56 ±1.75	2.68 ±1.19	2.01 ±0.99	0.62 ±0.08	0.67 ±0.07	0.62 ±0.08	192	218	335		34%	32%		72			86%
21d NC Avg	*n* = 2	-90	2.70 ±1.33	2.24 ±0.74	1.35 ±0.22	0.73 ±0.10	0.81 ±0.06	0.78 ±0.07	150	18	3							620 ± 210	0%
42d PO																			
42d PEG Avg	*n* = 6	-18	3.46 ±1.78	2.82 ±1.48	2.85 ±1.71	0.66 ±0.08	0.65 ± 0.07	0.67 ±0.08	211	280	210							2360 ±1100	99%
42d NC Avg	*n* = 6	-106	3.27 ±1.63	2.07 ±0.83	1.59 ±0.62	0.64 ±0.08	0.72 ±0.10	0.63 ±0.10	175	184	151							790 ±v 260	9%

Therefore, reinnervation after initial PEG-fusion occurs in the longer term by a combination of cellular mechanisms, such as: (1) immediate axonal reconnection by immediate (artificially induced) PEG-fusion; (2) distal axonal sprouting near the end organs, possibly producing hyperinnervation of some muscle fibers; and/or, (3) delayed (natural) regeneration via slower axonal outgrowth [4, 14, 15]. Both regeneration and PEG-fusion can produce specific or non-specific re-innervation of a target tissue and both can contribute to functional/behavioral recovery that is the best measure of PNI repair. Both regenerating axons, and *especially* PEG-fused axons, demonstrate extensive plasticity of PNS and CNS synapses and connections, perhaps by activation/de-repression of genes not active beyond embryonic stages [14, 15]. Data in support of, and consistent with, these statements are given in the following sections.

### PEG-fused PNAs to repair 0.5–1.0 cm segmental-loss PNIs in rat sciatic nerves exhibit atypical morphological and functional properties

1. Axolemmal and axoplasmic continuity that is rapidly (within minutes) restored as assessed by conduction of extracellularly recorded compound action potentials (CAPs) across all PEG-fused lesion sites (Fig. 3A) [4, 14, 15, 30, 32, 33, 57, 66]. Continuity is also confirmed by evoking CMAPs stimulated proximal to the PNA and recorded from muscle groups distal to the PEG-fused PNA (Fig. 3B) from 0 to 42 or more days post-operatively (PO) [30, 32, 33, 57]. Continuity is further confirmed by intra-axonal dye diffusion (Fig. 3C) and/or fast or slow transport of labeled proteins or tracers across the PEG-fused PNA [4, 30, 32, 33, 57, 66]. Finally, diffusion tensor images show continuous axonal tracts after PEG-fused repair, but not after Negative Control neurorrhaphy (see Fig. 5 of [66]).
2. Continuous maintenance from 0 to 42 days PO of distal segments of host or graft-donor myelinated, i.e., Wallerian degeneration is reduced or prevented, as assessed by gross anatomical inspection, TEM or IHC of PEG-fused PNAs (Fig. 2E–G; Table 2) and initial voluntary (albeit spastic) muscle movements (Fig. 3D) [4, 14, 15, 30, 32, 33, 57, 66]. At all PO times and in all observed segments of the nerve, axons in successfully PEG-fused nerves display rather normal ultrastructure with respect to myelin periodicity, tubulin and neurofilament arrangement, and mitochondrial ultrastructure. TEM analyses of cross-sections of PEG-fused PNAs and their proximal and distal host nerves from 0  to 42 days PO show many myelinated large-caliber axons that do not undergo WD. Successfully PEG-fused nerves show some increases in interspace area and changes in axonal diameter and myelination compared to intact control nerves, but overall gross morphology remains consistent from the proximal host through the graft and into the distal host region [4].
3. Continuous maintenance of nerve muscle junctions (NMJs) from 0 to greater than 84 days PO, as measured by CMAPs (Fig. 3B), TEM analyses, confocal immunohistochemistry (Fig. 3H–J), and counts of NMJs in innervated muscle fibers (Table 2). Furthermore, NMJs maintain muscle choline-o-acetyl transferase (ChAT) receptors neurofilament stained motor innervation (Fig. 3H–J) or sensory (carbonic anhydrase II: CAII) stained axons distal to a PEG-fused PNA [4, 14, 15, 30, 32, 33, 57, 66].
4. Continuous maintenance (e.g., minimal atrophy) of muscle fibers in PEG-fused PNAs similar to that seen in to Unoperated animals) as assessed by TEM and histological analyses that contrast with much degeneration/atrophy observed for NCs as assessed by the same analyses (Table 2) [4, 32, 33].
5. Behavioral functions that are initially spastic but whose coordinated activities are restored more rapidly and to levels significantly better than NCs. PEG-fused PNAs exhibit restoration of sciatic voluntary behaviors as assessed by of SFI scores that sometimes approach or equal that of Unoperated Control animals within 14–42-days post-repair (Fig. 3D, Table 2) [4, 30, 32, 33, 57, 66]. *As Brushart *[1]* has emphasized*, we hold that s*uccessful nerve repair after a PNI should always be defined by behavioral measures, such as the SFI*—*and not by axon counts or any other morphological or electrophysiological measure*. PEG-fusion non-selectively fuses more-proximal to more-distal axons at all repair sites. That is, PEG-fusion does not join motor to motor axons nor sensory to sensory axons, much less specific motor to specific motor axons (Fig. 4). For allografts, there is no axon-to-axon specificity between donor and host nerves, because the allograft has a different number of axons arranged in a different manner compared to proximal or distal host axonal segments. Nevertheless, behavioral recovery is often equal or superior for PEG-fused PNAs compared to PEG-fused single cuts or autografts (Fig. 3D, Table 2) [4, 14, 15, 30, 32, 33, 66].
6. Recovery of sensory functions for PEG-fused single cut sensory nerves in human clinical case studies is significantly better than primary neurorrhaphy without PEG-fusion [115]—which replicates what has been observed in rats (Fig. 3K) [30, 116].

**Fig. 5:**
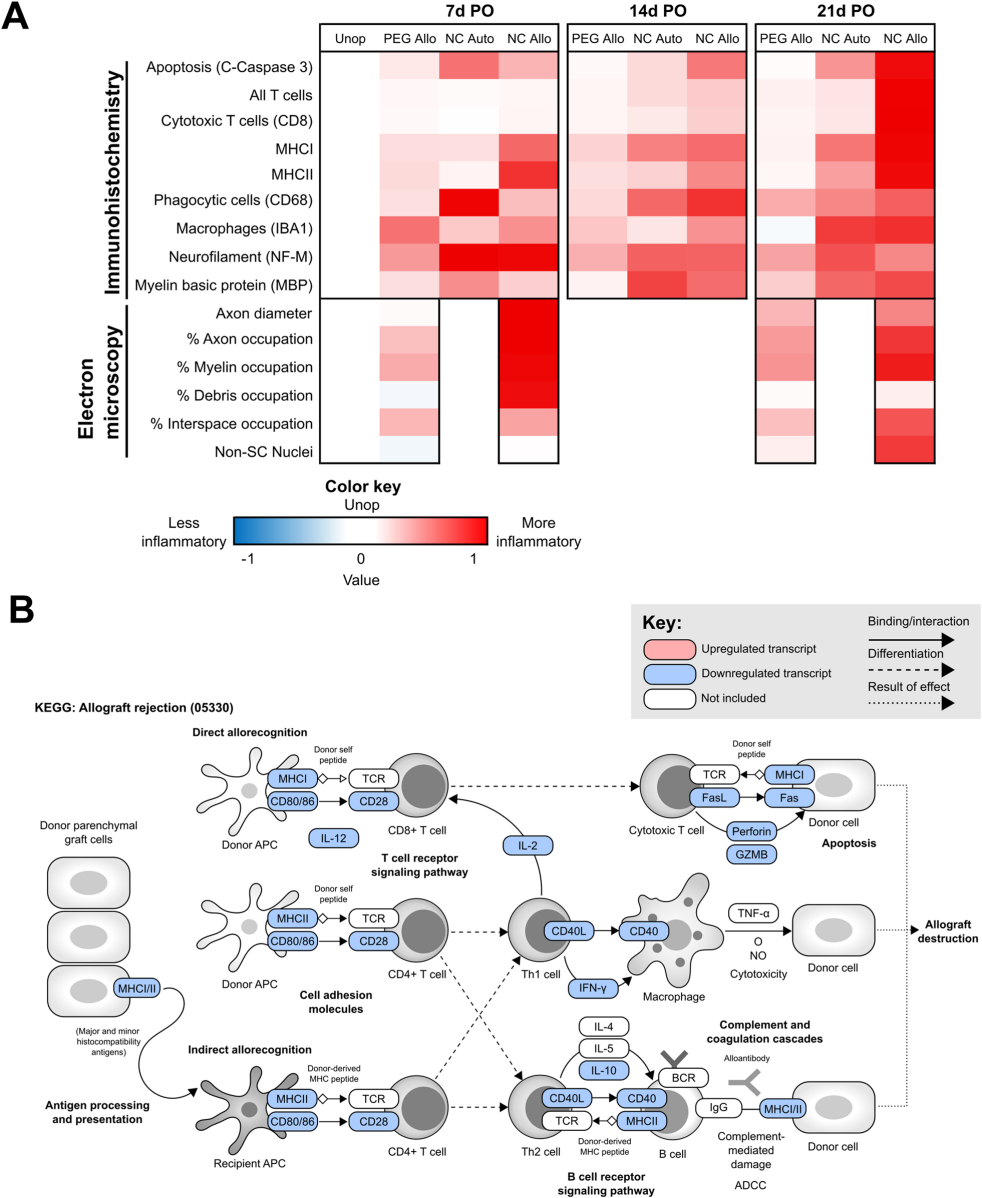
Overview of current immunological results via IHC, RNAseq, and RT-qPCR from Smith et al. [14, 15]. **A** Heat map of average measurements from immunohistochemical (IHC) stains (top 9 rows) and TEM images (bottom 6 rows) for Unoperated Control nerves, PEG-fused and Negative Control PNAs, and Negative Control Autografts at 7, 14, and/or 21 days PO. All values are relative to Unoperated Control values (white) and are normalized to a − 1 to 1 scale. Key: White to blue indicates a less inflammatory response; pink to red indicates a more inflammatory response. **B** Mapping of RNAseq data (differentially expressed genes compared between PEG-fused PNAs vs Negative Control PNAs sampled at 14-day PO) to the KEGG (Kyoto Encyclopedia of Genes and Genomes) Allograft Rejection pathway (2nd most highly enriched for downregulated transcripts in the PEG-fused PNA vs Negative Control PNA comparison via functional annotation) showing key molecules and processes involved in allograft rejection (ordered left to right in the diagram). Upregulated transcripts (pink tiles), downregulated transcripts (blue tiles), and transcripts that are part of the pathway, but not included in our list of DEGs (white tiles) are shown. No upregulated transcripts in PEG-fused PNAs mapped to the KEGG Allograft Rejection pathway. Solid arrows represent protein interactions, while dashed arrows pointing to or from T cells represent T cell differentiation into effector phenotypes. Dotted arrows point to a subsequent event. This diagram has been stylistically redrawn and modified from the original KEGG diagram for display purposes

Together, these data summarized in 1–6 above strongly support the basic scientific concept and clinical promise of PEG-fusion for repairing singly cut PNIs—and there is good reason to believe data in 1–6 above will hold for clinical use of PEG-fused PNIs to repair segmental-loss PNIs.

*In contrast, NC PNAs that are not PEG-fused do not exhibit any the phenomena 1–6 above for 7–42-days PO* (Figs. 2 and 3, Table 2). NC PNAs immediately lose continuity from the proximal, graft, and distal nerve segments assessed by electrophysiology (compound action potentials (CAPs), CMAPs) and dye diffusion. Axons in the graft and distal segments undergo Wallerian degeneration, and NMJs become denervated within 7 days PO. Muscle fibers undergo significant atrophy and often have central nuclei from 14 days to 42 days PO. There is no functional recovery by 42 days PO [4, 14, 15, 30, 32, 33, 66].

Interestingly, these remarkable PEG-fused data are obtained without any immune-suppression or tissue-matching, suggesting that PEG-fusion of PNAs alters the immunogenicity of PNAs. As discussed in subsequent sections, when compared to non-fused PNAs, RNA-Seq and IHC of PEG-fused PNAs demonstrated that PEG-fused PNAs had reduced T cell infiltration, reduced macrophage infiltration, reduced MHC expression, and upregulated extracellular matrix and adhesion molecules (Fig. 5) [14, 15]. As discussed in the previous section, PNAs are already comparatively less immunogenic than other transplanted tissues. This reduced immunogenicity appears to be further reduced by PEG-fusion and exposure to PEG per se, as discussed in subsequent sections.

Table 2 compares morphometric data (means ± SD or %) at three different PO times for repair of sciatic nerve segmental-loss PNIs by PEG-fusion PNAs and NC PNAs. Data from recently published papers [4, 32, 33] consistently demonstrate that: (1) Animals with PEG-fused single-cuts or allografts recover lost functions more rapidly and completely compared to NCs as assayed by the SFI in column 2. (2) Animals with PEG-fused sciatic nerves maintain axons, NMJs, and muscle fibers at all PO times, while NC preparations undergo Wallerian degeneration, denervation, and muscle fiber atrophy, as assessed by morphometric measures in columns 4–20. (3) NC sciatic nerves have smaller diameter axons that regenerate into the distal stump by 21-day PO and a few regenerating axons reach denervated muscles by 42-day PO. (4) Measures of g ratios and axon numbers are more variable and do not correlate well (p > 0.05) with SFI scores, as reported for regeneration by axonal outgrowth from proximal stumps.

#### PEG-fused PNAs to repair 0.5–1.0 cm segmental-loss PNIs in rat sciatic nerves exhibit atypical synaptic and other CNS and PNS plasticities to restore voluntary behaviors

After segmental-loss PNIs of 5–10 mm lengths in host Sprague Dawley rat sciatic nerves, PEG-fused PNAs from other donor wild type Sprague Dawley rats restored lost behavioral functions within 14–42-day PO (Fig. 3D) when neither host nor donors were tissue-matched or immune-suppressed. This behavioral restoration was almost-certainly by extensive PNS and CNS synaptic plasticities and collateral outgrowths, some of which are typically observed in adult mammals and others more typically restricted to embryonic stages of neuromuscular growth and innervation PO [4, 14, 15, 30, 32, 33, 66]. For example, PEG-fusion immediately preserves spinal motoneurons, changes their peripheral connectivity, and alters dendritic organization (Fig. 4). This spinal reorganization may contribute to the remarkable behavioral recovery that is not present at the time of axonal repair, but develops in the following weeks. CNS and PNS post-natal behavioral recoveries in response to PNS mis-wirings occur by training or exercise regimens and environmental enrichments [117]. Extreme levels of possible CNS rearrangements have been studied in rotations of sensory connections from the back and belly skin in frogs to produce changes in voluntary behaviors [118]. All these data suggest that spinal and supra-spinal CNS plasticities can play a role in pattern relearning for motoneurons that survive PNIs—and provide evidence of alternate pathways for restoration of function independent of reinnervation specificity.

In addition to these CNS plasticities, alterations in peripheral synapses (PNS plasticities) were also commonly observed after PEG-fusion repair. NMJs after sciatic severance or ablation followed by PEG fusion exhibited changes consistent with an environment of partial denervation and increased synaptic activity [4]. While some NMJs remained normally innervated (Fig. 2H–J), Terminal Schwann cell activation and process extension were seen in denervated NMJs at early timepoints (7–21 days). Early hyperinnervation followed by synapse elimination was also commonly observed after PEG-fusion, consistent with an environment of increased synaptic activity commonly seen after partial denervation. In contrast, no muscle fibers in NC PNAs were innervated from 7 to 21 days post-operatively, and *very* few NMJs were innervated at 42 days post-operatively compared to almost 100% innervation in PEG-fused PNAs (Table 2) [4].

PEG-fusion repair of some axons in a donor PNA and host distal nerve does not prevent natural regeneration of non-fused axons by outgrowths from surviving host stumps proximal to the PEG-fused PNA for at least 42-day post-injury. However, current data show that such outgrowths add little or nothing to the recovery obtained by surviving PEG-fused axons in PNAs at 42-day PO (Fig. 3D), and perhaps for many weeks thereafter in a few animals studied for longer PO times [4]. It is possible that such a second wave of regenerating outgrowths might be obscured by continued improvement from PEG-fused axons due to CNS and/or PNS plasticities and collateralization of fused axons.

Assessing the contribution of collateralization to functional recovery in longer term studies is complex. In peripheral nerve regeneration without axonal fusion, recovery of function is due to two major axonal processes: axonal outgrowth and collateralization of regenerated/ing axons. Axonal fusion adds at least two additional processes: fused axons and collateralization of fused axons. Unfortunately, there is no easy method to definitively disentangle how each of these four processes individually contribute to functional recovery, further complicated by the several branch points distal to a PNA and the possible contributions of central/peripheral plasticities.

In brief, PEG-fusion of PNAs must produce its dramatic functional/behavioral recovery by activating peripheral and CNS synaptic and other plasticities, quite possibly to a much greater extent than most neuroscientists currently believe to be possible [3, 30, 66, 119]. As noted [3, 4, 7, 14, 15], this systems level adaptation may include a profound reorganization that restores a normal pattern of behavior (‘multiple realizability’ [117]) and de-repress genes normally not active beyond embryonic or early postnatal stages. Trophic substances in the periphery, spinal cord and higher brain centers may help direct such re-organizations. Finally, extensive mis-connections in PEG-fused PNAs may evoke extensive synaptic plasticities and CNS and PNS axonal or dendritic outgrowths and rewiring to produce extensive behavioral recoveries.

#### PEG-fused PNAs that repair 0.5–1.0 cm segmental-loss PNIs in rat sciatic nerves exhibit atypical immunological properties

Two recent studies [14, 15] have demonstrated that successfully PEG-fused PNAs that are not tissue-matched or treated with systemic immunosuppressive drugs exhibit an *atypical* immunosuppressive microenvironment (Fig. 5A, B). Morphological, functional, immunohistochemical, and transcriptional analyses showed that many innate and adaptive immune responses that typically reject allogenic tissues were significantly attenuated in PEG-fused PNAs and do not produce functional rejection.

TEM data showed that PEG-fused PNAs maintained many large diameter axons (> 3 µm) that were well-myelinated by Schwann cells from 7- to 42-day PO [4, 14, 15]. The persistence of myelinating Schwann cells within PEG-fused PNAs suggested that many donor Schwann cells were not rejected at any post-operative (PO) time from 0 to 42 days. In contrast to NC PNAs that were not treated with PEG and that were rejected within 14–21-day PO, PEG-fused PNAs did not exhibit collapsed hollow Schwann cell basal laminae or degradation of epineural sheaths and blood vessel basal laminae at 21-day PO. lHC analyses showed that T cell and macrophage infiltration, phagocytic activity, and expression of major histocompatibility complex (MHC) I and II glycoproteins necessary for antigen presentation were significantly reduced in the intra-fascicular mid-graft regions of PEG-fused PNAs by 21-day PO [15]. In addition, PEG-fused PNAs had consistently reduced apoptotic activity as assessed via cleaved Caspase 3 immunostaining from 7- to 21-day PO (Fig. 5A).

Host T cells require chemotactic signals to infiltrate allograft tissues, and a combination of antigen interactions, cytokines, and co-stimulation to fully activate in response to donor cells [35]. Compared to NC PNAs, PEG-fused PNAs had consistently low T cell infiltration (Fig. 5), significantly lower at 14-day PO [15]. The reduction in T cell infiltration coincided with significantly reduced gene expression of key cytokines and chemokines involved in T cell trafficking and Th1 effector cell polarization, such as C–X–C Motif Chemokine Ligand 11 (CXCL11) and Interferon Gamma (IFN-γ) in PEG-fused PNAs [15, 120, 121]. An exploratory, bulk, non-specific, RNA sequencing study of the coding transcriptome of PEG-fused PNAs at 14-day PO showed molecular processes consistent with immunotolerance [14]. That is, compared to NC PNAs and Unoperated Control nerves, PEG-fused PNAs significant downregulated many T cell-associated gene transcripts (Fig. 5), including: (1) costimulatory receptors such as CD28; (2) Interleukin 2 Receptor Alpha (IL2RA); (3) cytokines involved in inflammatory Th1 cell polarization such as Interleukin 2 (IL-2) and Interleukin 12B (IL-12B); and, (4) transcriptional factors necessary for T cell activation such as GATA3 and T-box 21 (TBX21) [14, 122–125]. These results suggest either direct or indirect inhibition of T cell migration as well as activation in PEG-fused PNAs, which might be produced through several different mechanisms.

Reduced T cell activity might be due to an “immunocamouflage” effect produced in donor axon-Schwann cell units that have received host proteins and/or MHC molecules as a result of PEG-fusion. Nearby host T cells as well as host antigen presenting cells might not respond to axon-Schwann cell units that present host antigens. Direct inhibition of inflammatory T cell activation and effector functions by suppressor cells such as Tregs that secrete the anti-inflammatory cytokine IL-10 might evoke a local immune-acceptance in PEG-fused PNAs [108]. Lack of CD28 co-stimulation in host T cells upon antigen recognition might render them anergic in PEG-fused PNAs, thereby preventing them from responding properly [126].

However, as summarized in Smith et al., 2020B [14] and Fig. 5B, transcripts commonly involved in Th1 cell suppression such as IL-10, Cytotoxic T-lymphocyte Associated Protein 4 (CTLA4), and CD274 (also known as Programmed Death Ligand 1 (PD-L1)) were also significantly downregulated [14]. CTLA4 and PD-L1 are immune checkpoint inhibitors that bind to CD28 and CD80, respectively, on T cells and are critical therapeutic targets for immunosuppression via costimulatory blockade [127, 128]. The significant downregulation of these transcripts suggested that they were not heavily involved in immunosuppression associated with PEG-fusion. Nonetheless, CTLA4 and PD-L1-mediated immunosuppression of T cell activation pathways in PEG-fused PNAs is an important potential mechanism that warrants further investigation at the protein level.

Macrophages are numerous in injured peripheral nerves and PNAs [69]. In addition to their role in debris phagocytosis and promotion of regenerative processes in peripheral nerves, macrophages also heavily influence the inflammatory environment and often contribute to allograft rejection [35]. Upon tissue injury, many macrophages initially adopt a pro-inflammatory “M1” activation state in which they produce inflammatory cytokines, such as IL-12, and high levels of nitric oxide (NO) that can damage nearby cells [129, 130]. M1 polarization is stimulated by IFN-γ from Th1 cells, while IL-12 partially drives Th1 cell polarization [122, 130]. Macrophages thereby engage in positive feedback loop crosstalk with T cells to promote inflammation, antigen recognition, and donor cell death. In immunotolerated tissues, the reverse situation is also possible in which anti-inflammatory “M2” macrophages and Tregs can induce each other’s activation states to suppress Th1- and M1-mediated inflammatory responses and promote wound healing [108, 131, 132].

PEG-fused PNAs in IHC studies (Fig. 5A) had significantly reduced macrophage infiltration and phagocytic activity via CD68 and IBA1 immunostaining by 14–21-day PO compared to NC PNAs [15]. At 14-day PO, this result coincided with significantly reduced expression of common M1 macrophage markers such as inducible nitric oxide synthase (iNOS), reduced expression of the inflammatory cytokine Interleukin 1β (IL-1β) often produced by macrophages, and increased expression of common M2 macrophage markers, such as Arginase 1 (ARG1) and Mannose Receptor C-type 1 (MRC1, also known as CD206) [14, 130, 132]. These results suggest that PEG-fused PNAs might have both a reduction in macrophage infiltration as well as a shift in macrophage polarization that may contribute to an immunosuppressive environment. It is possible that lack of axonal damage in PEG-fused PNAs produces alternative Schwann cell signaling mechanisms and, therefore, alternative macrophage polarization.

The cleaved form of Caspase 3 is a common executioner of Granzyme- and Fas-mediated apoptosis by cytotoxic CD8 T cells or natural killer (NK) cells in rejected non-neuronal allografts and PNAs [35, 133–135]. Superoxide and NO cytotoxicity from ischemia reperfusion injury in organ allografts have also been shown to induce apoptosis via Caspase 3 [136]. After single-transection injury in sciatic nerves, Schwann cells, macrophages, neutrophils, and other cells regularly undergo turnover via apoptosis throughout the degeneration and regeneration process [69, 71]. Compared to NC PNAs, cleaved Caspase 3 immunostaining was consistently reduced to near-unoperated nerve levels in PEG-fused PNAs by 21-day PO, while Granzyme B and FasL expression were reduced on average at 14-day PO [15]. These data (Fig. 5) [15] suggested that both WD-associated apoptosis and rejection-associated apoptosis were inhibited in PEG-fused PNAs and might contribute to their immune-acceptance and functional non-rejection.

MHCI/II protein expression was reduced in PEG-fused PNAs and a set of transcripts encoding essential antigen presentation machinery components were downregulated in PEG-fused PNAs [14, 15]. These downregulated transcripts included Transporter 2 (TAP2) protein, needed to transport antigen peptides from the cytoplasm to the endoplasmic reticulum [137], and transcription factors that drive MHCI expression [Nod-like Receptor C5 (NLRC5)] and MHCII expression [Class II Major Histocompatibility Complex Transactivator (CIITA)] [138, 139]. These results (Fig. 5) [14, 15] suggested that mechanisms for antigen presentation and immunogenicity were also suppressed in PEG-fused PNAs. Lisak et al. (2016) [72] demonstrated that MHCII expression in SCs depends on their myelination state [72]. Myelinating SCs do not express MHCII and do not respond to IFN-γ stimulation, while demyelinated SCs do upregulate MHCII [72]. Therefore, the maintenance of many viable myelinated axons in PEG-fused PNAs may inhibit SC expression of MHCII and render those SCs non-immunogenic. Similar mechanisms might be involved in reduced MHCI expression of SCs in PEG-fused PNAs. It is also possible that the soluble 3.35 kD PEG used to PEG-fuse PNAs [14, 15] in these studies could inhibit MHC expression in PNAs, as shown in other studies examining kidney allograft storage in organ preservation solutions containing soluble PEG before transplantation [140].

Although PEG-fused PNAs had reduced immune responses described above relative to NC PNAs (Fig. 5A), PEG-fused PNA immune responses were much greater than these immune responses in Unoperated Control sciatic nerves [15]. Cellular infiltration, MHC protein expression, and cytokine/chemokine expression were higher in PEG-fused PNAs, suggesting that the signals necessary for orchestrating innate and adaptive immune responses were not completely eliminated, but rather suppressed at the transcriptional or post-translational level. It is possible that physical barriers in the blood vasculature, basal laminae, or other extracellular matrix components in PEG-fused PNAs impede infiltration by those immune cells, and thereby limit donor or host immune cell activation or induce alternative activation of immunosuppressive T cell and/or macrophage phenotypes [141–144]. Future studies will require single-cell sequencing, flow cytometry, and additional in vivo analyses to provide greater clarity as to the cell-specific nature of differential gene expression, cytokine production, and specific regulatory mechanisms driving immunosuppression in PEG-fused PNAs.

### Summary of how typical and atypical characteristics of PNAs provide good PEG-fusion and Treg results

Morphological observations of PEG-fused PNAs coincide and are consistent with their observed reduction in innate and adaptive immune responses. For example, compared to NC PNAs that are not PEG-fused, PEG-fused rat sciatic PNAs display significantly reduced total T cell infiltration, macrophage infiltration, inflammatory cytokine and chemokine expression, MHC I and II expression, and consistently low apoptotic response by 21-day PO [14, 15]. Many data from PEG-fused sciatic PNAs resemble data obtained for NC *Auto*grafts or even Unoperated Controls and Sham-operated Controls. Normal morphologies of Schwann cells and myelin in PEG-fused PNAs at 0–42-day PO suggest that donor Schwann cells that are prominent immunological targets in rejected PNAs, are not rejected/tolerated in PEG-fused PNAs at 42-day post-lesion repair. Furthermore, PEG-fused PNAs have exhibited non-rejection/ immunotolerance for at least 84-day PO [4, 33] to 112-day PO [32]. That is, PEG-fused PNAs are a unique, unstudied example of immunotolerance/immune-quiescence or greatly reduced immunoreactivity within a non-immuno-privileged environment of viable allograft tissue. All these IHC, gene transcription, electrophysiological (CAPs, CMAPs), intra-axonal tracer, and neuromuscular morphological (diameters, g ratios, NMJ % innervation) and behavioral (SFI) data cited above consistently suggest that PEG-fused PNAs remain viable, functionally non-rejected, and probably immune-accepted from 0 to at least 112-day PO. In contrast, NC PNAs are non-viable and immune-rejected at 7–14-day PO and recover few, if any, lost behavioral functions at 42-day PO [1–4, 6–15, 30–33, 46].

In addition to endogenous reasons why the immune response of PNAs may be somewhat immuno-privileged (surrounded by an epineural connective tissue barrier, reduced expression of MHCI and II, over proliferation of Tregs that repress effector T cells) as discussed in a previous section, PEG-fused PNAs maintain axon viability by diffusion or axonal transport of host proteins into donor PNAs [3, 4, 14, 15, 32, 33]. Hence, many donor axonal segments axons in PEG-fused PNAs may be recognized more as host tissue, rather than as foreign tissue. Second, PEG itself may have some immunoprotective effects [14]. Thirdly, host SCs may rapidly replace donor SCs in the PNA [6]. That is, In PEG-fused PNAs, if SCs myelinating PEG-fused axons were to be rejected, the axon should completely demyelinate until a host SC could migrate to remyelinate it. However, successfully PEG-fused axons maintain their myelinated state indefinitely after transplantation [3, 4, 14, 15, 32, 33].

In brief, the typical goal for most non-neuronal allograft transplants is to replace the function of host organs or tissues by maintaining viable donor cells and non-cellular structures to immediately and continually perform the same function as the host organ/tissue (e.g., heart pumping of blood) [6, 12]. In contrast, the goal for PNA tissue transplants is atypical in that restoration of function is not expected and depends on host axons growing across the PNA to eventually re-innervate denervated host muscle, sensory or organ structures. Donor cells are not maintained in PEG-fused PNAs and PEG-fusion immediately repairs many cells (axons) in the PNA to create host/donor chimeras that rapidly acquire the immunological properties of host cells by rapidly re-establishing fast and/or slow axonal transport and/or cytoplasmic diffusion. Host Schwann and vascular cells rapidly infiltrate PEG-fused PNAs (and NC PNAs). Nerve cells and their axons may have less ability to evoke an immune response than other cell types and PEG itself has some immunoprotective effects [3, 4, 6, 14, 15, 32, 33].

### Problems of, and possible solutions for, PNA use and storage

Potential problems for use of PNAs to repair segmental-loss PNIs include WD of axons in the PNA, host/donor nerve immunological incompatibility, immunosuppressive medications for PNA maintenance that are currently (and extremely rarely) used, and logistics associated with the procurement and dissemination of viable human tissue.

#### Immunosuppression

Immunosuppression is one of the primary issues that has prevented, and continues to prevent, widespread use of PNAs to repair segmental-loss PNIs. Immunosuppression is required with PNAs to achieve full regeneration by axonal outgrowth equivalent to mixed autografts, but systemic immunosuppression carries risk for pathogens, cancers and systemic toxicity [145–148]. For example, a single case of a living related donor PNA reconstruction in a 1-year-old patient with immunosuppression resulted in symptomatic Epstein–Barr viral infection that required subsequent withdrawal of immunosuppression and potential increased lifetime risk of post-transplant lymphoproliferative disease [177]. The risks of immunosuppression are serious and are tolerated in quantity-of-life scenarios, such as life-saving organ transplantation, but not generally in cases of PNIs using PNAs that would be quality-of-life transplants. Immunosuppressive therapy is also expensive to administer and monitor [145–149]. For PNAs to be used more widely, reliable techniques and technologies for immunosuppression need to be developed that are safer, less expensive, and not resource intensive. While the level of immunotherapy required for PNAs may be less than that of traditional organ transplantation, this level has not been widely studied and the threshold for immunotherapy is not currently known.

Whether a PNA is used like a traditional nerve graft (as a scaffold for nerve regeneration), or combined with PEG-fusion, premature graft rejection could be a significant adverse event. The effectiveness of temporary systemic immunosuppression shows that long-term tolerance isn’t necessary for this temporary scaffold use of PNAs [27, 28, 149]. However, the time that the conventional PNA must be protected from the host immune response has not been determined. PEG-fused PNAs are not rejected for at least 5 months in rats, the longest time studied [15, 32, 33]. Hyperacute rejection may also occur despite immunosuppression and could necessitate pre-graft crossmatching for conventional use of PNAs. In initial human case studies, immunosuppression with a combination of anti-IL-2, tacrolimus, and azathioprine has been used until axonal regeneration presumably extended beyond the PNA. The immunosuppression was then withdrawn on the assumption that the axons and supportive cells now express host MHCs and do not require further immunological protection [148].

#### Potential for other adverse events

Aberrant axonal regeneration in segmental-loss PNIs might produce significant clinical dysfunction for locally immune suppressed or PEG-fused PNAs. As one possibility, neuromas are painful sensory fiber outgrowths that can occur with any PNI and are observed with autografts, conduits and decellularized allografts [150–152]. Neuromas are more common when the environment around a PNI becomes inflammatory, which is a yet-larger concern for a PNA. Aberrant sensory function is likewise more common with inflammation after segmental-loss PNIs.

#### Data showing successful PEG-fusion and local immunosuppression of PNAs in experimental animal models may outpace current clinical conditions

A supply of human PNAs is currently not available and current clinical surgical protocols often repair PNIs (especially segmental-loss PNIs) after many days post-injury when WD is well established or complete, thereby precluding axonal PEG-fusion. Such clinical protocols would require revisions. That is, the primary issue for surgical timing is that successful axonal PEG-fusion repair depends upon having viable host and donor axons. Fortuitously, there are promising methods in development to slow WD in stored PNAs and in distal host nerve tissue [153–159]—which would extend the timeline in which PEG-fusion could be applied post-injury. A combined PEG-fusion and local immunosuppression approach could partially mitigate this clinical issue. Furthermore, localized immunosuppression with a PNA might enable robust axon regeneration in cases when it is too late to apply PEG-fusion or in cases in which PEG-fusion is attempted but fails due to the considerable variability that can occur in a clinical setting. Finally, donor PNAs can be stored for at least 3–4 days without loss of PEG-fusion capability—and possibly 4 weeks without a loss of support for axonal regeneration by axonal outgrowth [160]. Hence, recovery, storage, and supply to surgeons could meet future possible demands.

### Clinical implications and commercial availability of PNAs to repair segmental-loss PNIs

#### Clinical implications

PNIs are a major source of disability today and major segmental-loss PNIs still have a relatively poor prognosis compared to other injuries, especially when one or more mixed motor/sensory nerve is involved [161–164]. PNIs occur at an estimated rate of 1.64–2.8% of civilian inpatient hospital trauma admissions and 15–25% of all wartime combat and non-combat injuries [165]. These percentages translate to an estimated 9,900 brachial plexus and major upper limb PNIs per year. The collective disability that results from these major PNIs is far greater than these low numbers might suggest [161, 162, 165, 166]. PNIs are typically *the* major source of disability associated with extremities that have sustained concomitant injuries to bone, vessels, and soft tissues. Despite significant progress in the treatment of injuries of bone, blood vessels, and soft tissues, relatively little clinical progress has been made in the treatment of major PNIs. Despite decades of research with growth factors, cell cultures, engineered conduits, etc., the gold standard for repair of major PNI is a primary repair (for a simple transection injury) or autograft nerve reconstruction (for segmental or “ablation” type injuries), with microsurgical technique just as it has been for the last 30 or more years [167–170]. The commercialization of conduits and acellular nerve allografts has been a helpful adjunct. However, the gold standard of cable autograft neurorrhaphy still persists and functional recovery from major segmental-loss PNIs still remains a slow and incomplete process for most adult patients and experimental animals [1–7, 64, 66, 167–170].

The most significant clinical problem with repair/reconstruction of PNIs is the unavoidable WD that dooms the injured recipient to a slow and incomplete nerve regenerative process and often results in significant muscle atrophy and even permanent loss of NMJs, temporary or permanent loss of protective sensation, and profound functional/voluntary behavioral disabilities [171, 172]. Autograft reconstruction of segmental-loss PNIs require that some level of further impairment is created by taking functional nerves from a region of lesser importance to rebuild those of greater importance [173]. Not only does this create an iatrogenic deficit that is typically sensory, since those are considered the most expendable nerve donors, but also may further impair nerve regeneration by providing only sensory-type Schwann cells with may provide less regenerative capacity for motor axons resulting in poorer motor recovery [174, 175].

#### Commercial availability

Allograft tissues of any type (including PNAs) are not necessarily immediately available in any circumstance. Tissue banks and organ procurement organizations (OPO’s) for cadaveric donor tissue require infectious disease screening, tissue and blood group matching when appropriate, and special permissions from donor families, recipients, and hosting medical centers. The use of living related donors may not require the participation of an OPO, but ideally involves an experienced transplant center that will still screen donors and recipient for infectious diseases, tissue/blood types, and has a transplant surgeon or nephrologist who will monitor the recipient and manage their immunotherapy. While the conventional use of PNAs is somewhat time dependent (up to approximately 7 days with cold storage in University of Wisconsin solution) [27], it is less time dependent than traditional organ transplants. Wider use of PNAs would require a demonstrated benefit and engagement from OPO’s, peripheral nerve surgeons, transplant centers and their personnel to be done on any significant scale. As described in previous sections, there are real risks associated with transplanted tissue, not to mention the complications that can arise from even “short” periods of immunosuppressive medications (kidney disease, malignancy, infectious diseases, etc.) [28]. Recovery of PNAs will require specialized training to expose, isolate and remove PNAs without damaging this delicate tissue and likely involve a team of donor surgeons with expertise in peripheral nerve surgery similar to how solid organs and even vascularized composite tissue allotransplants, such as hand and face transplants, are procured. It will also be important to develop qualifications for what nerves may be used given their proximity to surrounding traumatized tissue. Use of damaged nerve segments would be likely produce inferior outcomes.

## Conclusions and future directions

The availability of viable PNA’s may significantly change the reconstructive options for segmental-loss PNI’s in many ways. Tissue banks for PNAs could potentially allow for the procurement of topographically similar nerves with motor or sensory specific SCs and other supportive cell types to better promote motor and sensory axonal regeneration. Donor PNAs would prevent the need for host nerve procurement and further insult of the injured patient. In addition, successful PEG-fusion of PNAs produces rapid return of some immediate sensory and motor nerve functions and preservation of some NMJs and muscle fibers.

PEG-fused PNA and local immunosuppression technologies described herein potentially eliminate many of the problems associated with the use of conventional PNAs described in previous sections of this review, but create other problems such as a need to repair segmental-loss PNIs within 24–48 h rather than three or more days after the injury. PEG-fused PNAs uniquely produce “immune-acceptance/ functional non-rejection” in the absence of tissue-matching and immunosuppression—a result that may be further enhanced by local immunosuppression. Further refining these advances to promote some immediate behavioral recovery without the need for systemic immunosuppression in small mammal experimental models—a result that may be further enhanced by local immunosuppression. Translating these findings to larger animal models that are more clinically relevant and further refining these advances to promote some immediate behavioral recovery without the need for systemic immunosuppression are realistic goals that would provide significant benefits over any other widely and clinically available technology.

Future research might extend the time in which axonal PEG-fusion can be reliably achieved following PNIs. For segmental-loss PNIs, an extended time to irreversible WD is needed for both stored PNA grafts and host distal anucleate axons. Our unpublished data show that PNAs can be stored for at least 3 days after harvest and still achieve axonal fusion. Ideally, when surgeons first assess a PNI and determine that the patient would be a candidate for axonal fusion, it would be advantageous to have an implantable device (or drug) that would slow WD and thereby increase the window of time in which axonal fusion could be applied—which our preliminary studies indicate is at least 36 h post injury. Prolonging this time frame even by 50% could have a tremendous impact. The process of WD has been studied and several promising lines of investigation for slowing this process have already been discovered [154, 155, 163, 164]. We are, therefore, optimistic that slowing WD both in vivo and in stored grafts is an achievable goal.

## Data Availability

Not applicable.

## Abbreviations

APC: Antigen presenting cell
ARG: Arginase 1
BHRP: Botulinum-conjugated horse radish peroxidase
BNB: Blood nerve barrier
CIITA: Class II major histocompatibility complex transactivator
CAII: Carbonic anhydrase II
CAP: Compound action potential
CD: Cluster of differentiation
ChAT: Choline-*O*-acetyl transferase
CMAP: Compound muscle action potential
CNS: Central nervous system
COL1A1: Collagen type I alpha 1 chain
CAP: Compound action potential
CsA: Cyclosporine A
CTLA4: Cytotoxic T-lymphocyte associated protein 4
CXCL11: C–X–C motif chemokine ligand 11
DCHS1: Dachsous cadherin-related 1
DC: Dendritic cell
DRG: Dorsal root ganglion
GATA3: GATA binding protein 3
GFP: Green fluorescent protein
IHC: Immunohistochemistry
IFN-γ: Interferon gamma
IL-12B: Interleukin 12B
IL-1β: Interleukin 1β
IL-2: Interleukin 2
IL2RA: Interleukin 2 receptor alpha
iNOS: Inducible nitric oxide synthase
LABC: Lateral antebrachial cutaneous nerve
LC: Langerhans cell
MABC: Medial antebrachial cutaneous nerve
MB: Methylene blue
mHA: Minor histocompatibility antigen
MHC: Major histocompatibility complex
mHC: Minor histocompatibility complex
MRC1: Mannose receptor c-type 1
NC: Negative control
NFAT: Nuclear transcription factor activated T cell
NK: Natural killer cell
NMJ: Nerve muscle junction
NLRC5: Nod-like receptor C5
NO: Nitric oxide
OPO: Organ Procurement Organization
PD-L1: Programmed death ligand 1
PEG: Poly(ethylene glycol)
PEGNB: Poly(ethylene glycol) norbornene
PN: Peripheral nerve
PNA: Peripheral nerve allograft
PNI: Peripheral nerve injury
PNS: Peripheral nervous system
PO: Post operatory
SC: Schwann cell
SFI: Sciatic functional index
TCR: T cell receptor
TBX21: T-box 21
TEM: Transmission electron microscopy
THBS1: Thrombospondin 1
Treg: Regulatory T cell lymphocyte
WD: Wallerian degeneration
